# Design a Database of Italian Vascular Alimurgic Flora (AlimurgITA): Preliminary Results

**DOI:** 10.3390/plants10040743

**Published:** 2021-04-10

**Authors:** Bruno Paura, Piera Di Marzio, Giovanni Salerno, Elisabetta Brugiapaglia, Annarita Bufano

**Affiliations:** 1 Department of Agricultural, Environmental and Food Sciences University of Molise, 86100 Campobasso, Italy; e.brugiapaglia@unimol.it (E.B.); annaritabufano84@gmail.com (A.B.); 2 Department of Bioscience and Territory, University of Molise, 86090 Pesche, Italy; piera.dimarzio@unimol.it; 3 Graduate Department of Environmental Biology, University “La Sapienza”, 00100 Roma, Italy; salerno868@gmail.com

**Keywords:** wild edible plants (WEPs), database, AlimurgITA, ethnobotany, Italy

## Abstract

Despite the large number of data published in Italy on WEPs, there is no database providing a complete knowledge framework. Hence the need to design a database of the Italian alimurgic flora: AlimurgITA. Only strictly alimurgic taxa were chosen, excluding casual alien and cultivated ones. The collected data come from an archive of 358 texts (books and scientific articles) from 1918 to date, chosen with appropriate criteria. For each taxon, the part of the plant used, the method of use, the chorotype, the biological form and the regional distribution in Italy were considered. The 1103 taxa of edible flora already entered in the database equal 13.09% of Italian flora. The most widespread family is that of the Asteraceae (20.22%); the most widely used taxa are *Cichorium intybus* and *Borago officinalis*. The not homogeneous regional distribution of WEPs (maximum in the south and minimum in the north) has been interpreted. Texts published reached its peak during the 2001–2010 decade. A database for Italian WEPs is important to have a synthesis and to represent the richness and complexity of this knowledge, also in light of its potential for cultural enhancement, as well as its applications for the agri-food system.

## 1. Introduction

One of the most famous aphorisms attributed to Hippocrates of Cos (“let thy food be thy medicine and medicine be thy food”) underlines the strong correlation existing between food and its curative capacities whose effects are synergic, not separable in their overall actions [1,2,3,4,5,6]. It is therefore likely that some plants, widespread in the tradition of many cuisines and which are still eaten every day, originally introduced because of their curative properties, then—over time—might have become staple foods in the local cuisines. For example, many species of the *Allium* genus, as well as many species with a bitter taste (e.g., *Cichorium intybus*), show excellent curative properties, with their use being testified in many civilizations since ancient times (the Ebers papyrus, an Egyptian medical text dated 1550 B.C., includes long descriptive sections about the medicinal use of these plant species).

The dual value of the use of plants as foodstuff/medicine—expressed today through the words “phytoceuticals” [7,8] and “nutraceutics” [9]—therefore, has accompanied the history of mankind for millennia through the accurate choice of species to be gathered and domesticated [10]. As a consequence, each diet is strongly related to the environment and to what is available there, i.e., to its biodiversity.

According to [11] there are between 300,000 and 500,000 plant taxa, of which about 10% have proved to be edible. As stated by [12] there are 350,000 plant species in the world, and about 80,000 of them are edible by humans. Based on FAO data [13] there are about 6000 plant species which have been cultivated from the beginning of time to the present day for food purposes; only about 200 of them are cultivated on a significant scale, of them only eight approximately (barley, beans, peanuts, corn, potatoes, rice, sorghum, and wheat) are crops which provide more than 50% of our daily calorie intake.

In Europe [14,15,16], the use of about 1600 wild species is known, accounting for about 13% of all its flora; on the other hand, in the eastern Mediterranean region, the list includes as many as 2300 taxa, of which 1000 are used solely at local level [17,18].

The Food and Agriculture Organization (FAO), has estimated that, over 100 million people in the EU consume wild plants [19], the latter being a part of people’s diet around the world and playing an important role in the Mediterranean diet.

In the case of Italy, it has been estimated that in 2013 the total number of Wild Edible Plants (or WEPs) includes 828 taxa, amounting to about 12% of the whole Italian flora; this estimate—apart from wild plants—includes those consumed as food, aromatic herbs, and liqueurs [20]. More recently, 276 WEPs taxa used in traditional vegetable mixtures have been analyzed also from an ethno-pharmacological relevance perspective [21]. The studies about the various regions or more limited territories thus seem to provide sufficient knowledge regarding wild edible plants.

Important international institutions (FAO, WHO, UNESCO, WWF) have taken an interest (directly or indirectly) in ethnobotany and alimurgy, attempting—through the recovery of popular traditions—to provide a meaningful contribution to resolving humanitarian problems such as hunger in the world [13,22], the search for new biologically active molecules for the cure of diseases [23], safeguarding minority cultures, protecting biodiversity, and taking care of the landscape [24].

In Italy, during the same period, this cultural trend has led to a significant increase in ethnobotanic studies. According to [25] this type of studies reached its peak between 1991 and 2000, then continued to grow in later years. Also the number of papers written on the subject of alimurgy—as will be detailed in the following sections—has shown a similar upward trend over the past 30 years.

What is the cause of such increasing interest? We consider that, to sum up, it may be associated with five reasons:

1. More attention being paid to salutogenesis also through the rediscovery of food, diets and dishes closely related to specific regions. This process is gradually helping to fill a methodology gap in the relationships between diet and use of wild plants as food [26]. For example, in the Mediterranean diet [27,28], according to the definition by the American epidemiologist/biologist Ancel Keys [29], a fundamental component comes from plant foods, including those spontaneously available in nature. *Salutogenesis*, a term coined by [30], is a subject whose aim is to foster the development of health through a process of discovery and use of individual health sources, also at environmental level. The Mediterranean diet is a nutritional model inspired by food models widespread in several countries of the Mediterranean area, founded on the habitual consumption of specific foodstuffs including mainly cereals, fruit, vegetables, grains, olive oil, rather than red meat and animal fats (saturated fats), with a lower percentage of fish, white meat (poultry), legumes, eggs, dairy products, red wine, and sweets. In 2010 UNESCO acknowledged it as a protected asset and added it to the list of oral and immaterial world heritage.
2. Opportunity for sustainable agriculture [31]. The increased knowledge of WEPs could have a useful impact on cultivation in marginal areas with a low use of energy inputs, which would have a positive impact on several levels, including the complexity of agroecosystems, increasing their biodiversity. There is also a very close link between WEPs and the cereals, fruits and vegetables that are part of our diet. This link is represented by CWR (crop wild relatives) the wild ancestors of cultivated plants which are recognized as having a strategic role for the conservation and sustainable use of plant resources in agriculture [32] also within the future scenario of climate change [33,34,35].
3. Creation of new product ranges for the food industry, also including functional foods, fresh or processed food with beneficial and protective properties for the body which go beyond mere nutritional properties [36,37,38,39,40,41,42,43].
4. Enhancing physical and cultural specificities of a given region, in line with the concept of ‘terroir’. Terroir, is an untranslatable French word which originally comes from vine growing, to define the interaction between physical factors (soil, exposure, climate), crops (vine varieties), and culture (vine cultivation), also including the product consumers.
5. Awareness of the importance of preserving and keeping the complex of ethnobotanic traditions alive; the recovery of collective knowledge, in itself, has an intrinsically high cultural value as historical memory of civilizations (farmers and shepherds) which are now lost or have undergone deep changes becoming impoverished in terms of passing on know-how. Over the past few years there has been an increasing transfer of ethnobotanical knowledge, not between elements of the same culture but rather from one society to another, consisting essentially of experts on the subject or of enthusiasts (foragers).

Despite the large number of data published on WEPs in specialized literature, what still seems to be lacking is a synthetic and systematic framework of actual knowledge to show the actual relevance of alimurgic species as an asset, using stringent criteria and assessments over a significantly long period of time. Evidence of this is the fact that several databases have been produced—available for public reference—but none of them specific for WEPs. A general list, still mostly valid, is included in Table 1.

Despite this increase in scientific papers, the question remains open of whether knowledge of WEPs is considered sufficient for Italy and for each of its administrative regions.

Hence the need to compile a database of Italian alimurgic flora aimed at providing a comprehensive knowledge framework, with a view to covering the wealth and complexity of this knowledge, also in the light of its potential for cultural enhancement, as well as of its applications for the agro-food system.

Today Italy would need a free access database which can collects—comprehensively and systematically—the wealth of knowledge regarding alimurgic flora in the country. Within the current limited framework, it is worth mentioning the Centro Etnobotanico Toscano (CET), established in 1999 with headquarters at the Department of Agricultural, Food, and Agro-environmental sciences at the University of Pisa, whose initial purpose was to serve as regional and national point of reference for disseminating information regarding ethnobotany. This archive, which is not accessible online for the general public, has collected, in Tuscany, more than 70 ethnobotanic publications and created an online database with all their contents [44,45,46]. There are currently 732 classified ethnobotanic species in Tuscany, mainly used for medicinal purposes (542 plants) and as food (366). In the same year, a paper was published announcing the establishment of a structured ethnobotany database for the Liguria region [47], which is not yet available for reference by the general public.

Later on, in 2003, the Project called RUBIA [48] was started, the first ethnobotanic research work funded by the European Commission, whose purpose was to safeguard the ethnobotanic heritage in several Mediterranean countries (Albania, Algeria, Cyprus, Egypt, Italy, Morocco, and Spain), regarding the botanical aspects of the elements under investigation, as well as all related traditional items and technologies [48,49]. The main results concern a total of 985 species catalogued, of which 406 taxa have medicinal use [50] and 294 wild food plants [51] were documented. Although this work constitutes the first comparative study performed with ethnobotanical data collected by a coordinated methodology in the Mediterranean area, overall data for alimurgic species are scarce.

In in his compendium “Usi e tradizioni della flora italiana” [25] mentioned 526 taxa used as food and as aromatic plants in Italy, on a bibliographical basis. 

A further attempt to quantify the Italian alimurgical flora was recently made, on a limited number of texts, by [52].

The data presented here will thus refer to the AlimurgITA database which has been created at the Botanic Laboratory of the University of Molise, whose structure has been developed in order to rapidly create an interface with the major portals dedicated to Italian flora (Dryades, Acta plantarum) for user-friendly open reference. The AlimurgITA database will be available for consultation in the coming months on the website of the University of Molise.

The final aim of our study is to systematically collect the large amount of data regarding plants which occupy or used to occupy a significant position in the area of Italian alimurgic botany.

This database could be serve as be a tool for work and dissemination containing information which is already available literature (including both science disciplines and the humanities), which in the future could be added to those made available by research currently underway. 

The use of WEPs has become known in Italy under the name ‘alimurgy’, now of current use not only in the strictly scientific environment, but also among many enthusiasts of this subject matter. Even though the term is often quoted, its meaning is not yet sufficiently clear: it is therefore essential, in our view, to provide a few clarifications.

### Clarifications on the Word ‘Alimurgy’ (Alimurgia)

This neologism was introduced by Giovanni Targioni-Tozzetti, author of the work by the same name dated 1767 [53]; strangely enough, the word “*alimurgia*” is mentioned only twice in the text, without clarifying its etymological root: the author was presumably certain that the learned audience of readers for which the work was intended would understand its hidden meaning. With an innovative editing structure and rigorous scientific method, the publication “*alimurgia*” was mainly focused on agriculture, ranging from the knowledge of the climate in the Tuscan region, to the choice of cereal seeds—the most suitable for the various territories—also including a study of wheat leaf rust, a disease which he was the first to discover and study. The use as food of wild species during famines, or of those to be introduced profitably in crops, therefore appears to be of marginal interest. Even though it was initially mentioned by Targioni-Tozzetti, this use was never dealt with later, possibly due to the work being incomplete, since it was expected to include a second volume which was actually never published [54,55]. This is why there was no discussion about edible wild plants; the topic had been dealt with, only in part, in one of his earlier works [56], where several spontaneous plants are examined which—added to wheat flour—could be successfully used for bread-making.

After one century and a half of oblivion, the word alimurgy was used again by [57], who added the prefix *phyto*- to the word alimurgy, then specified its area of interest, namely the use of wild species during periods of food shortage. At the same time, he provided an interpretation of the word ‘*alimurgia*’, a combination of the Latin words *alimentum* (food) and *urgeo* (to press, to urge). According to [58] the suffix –*urgia* comes from the Greek word *érgon* (work).

The word alimurgy was later redefined by Mattirolo who confined it from its original and broadest meaning related to practices aimed at a greater efficiency of the agricultural system, aimed at preventing or containing food shortages in the future, to the one limited to wild species alone. From the time of Mattirolo onwards, possibly also due to the confidence in this prominent author or to the limited knowledge of the work by Targioni-Tozzetti, the word “alimurgy” has taken on the meaning, which we commonly attribute to it today.

## 2. Materials and Methods

The setting up of the Database AlimurgITA has made it necessary to introduce a few conditions as regards the choice of data to be entered.

First of all, only vascular plants have been considered, excluding from the calculation of alimurgic species fungi and lichens (e.g., *Cetraria islandica* used during a famine in the Veneto region in 1916) [59], a subject of limited consideration in most texts of an ethnobotanic nature. The results obtained were thus extremely partial, therefore insufficient for a comparison between plants and on an ethnobotanic basis.

In AlimurgITA database, all the data contained in the selected texts according to subsequently specified criteria, have been included, as considered valid a priori. 

As part of the index of taxa included in the various works published, a choice was finally made of the strictly alimurgic one, that is to say plant taxa—autochthonous or alien then becoming naturalized—where one or more parts was considered edible in some areas of the Italian territory studied from an ethnobotanical perspective.

This means that casual alien and archaeophyte species [60] have not been considered alimurgic, i.e., those which are found only occasionally in very small parts of the territory and all the cultivated varieties which are frequently found in the lists of numerous publications on the subject of alimurgy. By the same token, according to the status defined by [60] and most recently updated according to *Acta plantarum* [61], the records do not include taxa reported by mistake, those which are either no longer recorded, absent or doubtfully occurring. 

On the other hand, the database includes species found both in their spontaneous and cultivated state (e.g., olive, chestnut, vine, hazelnut, fig, white mulberry) even though domesticated forms are widely dominant.

The nomenclature of the taxa mentioned refers to the checklist by [62] and [60]. Where necessary, the binomial according to the Word Flora online [63] is shown in brackets with the exclusion of nominal subspecies.

Several critical taxa from a taxonomy perspective, for example *Taraxacum officinale* and *Rubus hirtus*, have been taken into account as a group; in the case of *Portulaca* the choice has been made, on the contrary, to keep it as *P. oleracea* s.l., in view of the impossibility to refer back the records to the taxa into which it is divided. A similar criterion was applied for *Thymus serpyllum*.

**Figure 1:**
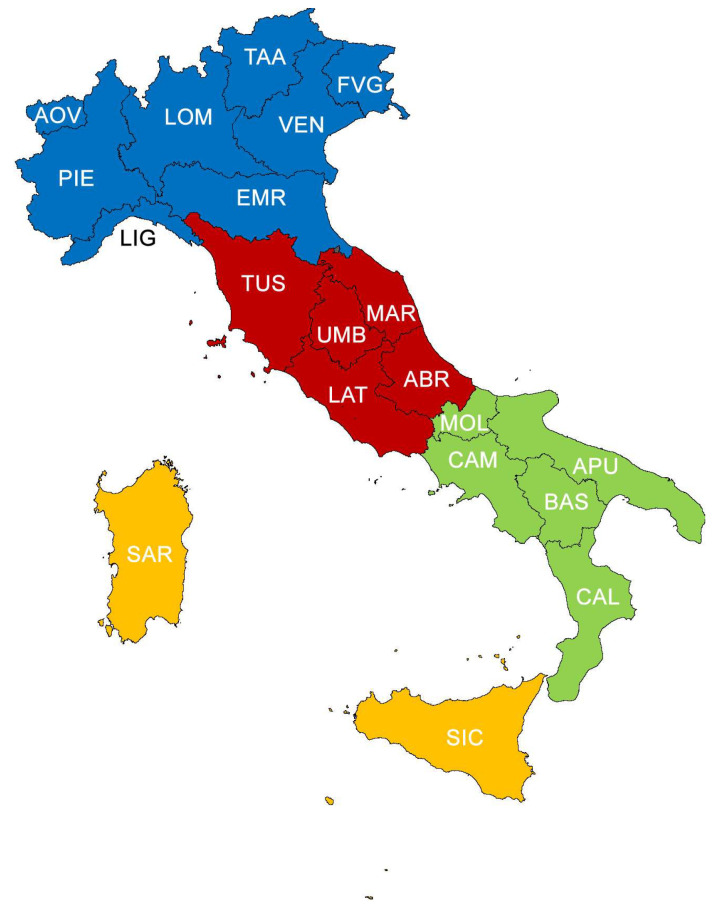
Division of Italy into sectors (North—blue, Centre—red, South—green, major islands—orange). Abbreviations of the administrative regions: AOV = Aosta Valley, PIE = Piedmont, LOM = Lombardy, TAA = Trentino-Alto Adige, VEN = Veneto, FVG = Friuli-Venezia Giulia, LIG = Liguria, EMR = Emilia Romagna, TUS = Tuscany, UMB = Umbria, MAR = Marche, ABR = Abruzzi, LAT = Latium, CAM = Campania, MOL = Molise, APU = Apulia, BAS = Basilicata, CAL = Calabria, SIC = Sicily, SAR = Sardinia.

For a comparison on a national scale, an estimate has been made of the reference flora for Italy using data from the checklist of spontaneous vascular flora in Italy [62] and of alien flora in Italy [60]. More specifically, from the first list, the taxa present in Italy have been included, whereas extinct taxa have been deleted, as well as those no longer found and those reported by mistake, amounting to a total of 7582 taxa; the second list does not include casual taxa in all Italian regions, those which are extinct, no longer found and reported in error, amounting to a total of 844 taxa. On the contrary, the database still include alimurgic taxa whose presence in the region was considered uncertain, thus implicitly validating the identification completed. Starting from these 8426 taxa, moreover, a list has been compiled on the basis of species included in the database AlimurgITA, of alimurgic taxa potentially present in every Italian region, to be associated with species actually listed in the census both at regional and geographical sector level (North, Centre, South, Major islands) (Figure 1).

As regards modes of consumption, the species used exclusively for herb teas, infusions and decoction have not been included, because their purpose is mainly associated with their medicinal properties rather than with the food sector [64]. On the contrary, the database includes species used for preparing liqueurs because the latter are often considered as an integral part of the meal, according also to [64].

In order to also save all data related to excluded taxa, their list is included in the Supplementary Materials Table S1.

A critical discussion about the alimurgic Italian checklist is therefore postponed to the next scientific paper. 

A further aspect then concerned the choice of texts to be entered. Over the past few years, alongside the growing interest in WEPs, there has been a proliferation of writings, both of a scientific nature and for the general public. The chosen window of observation included the past one hundred years (1918–2021), taking as starting point the year 1918—i.e., when *Phytoalimurgia pedemontana* was published [57]—because we consider that to be a ground-breaking text for modern botanical alimurgy.

It did not seem appropriate to enter this substantial amount of data uncritically, therefore we decided to choose published texts which met the following two criteria:

1. Publications in scientific journals or books;
2. Publications also in non-scientific journals, provided that they had been compiled by authors referenced for alimurgic subjects.

These two criteria are also associated with a reference to clearly defined territories, up to the national scale; the idea was to follow a specific ethnobotanic approach, highlighting contributions which established a close relationship between WEPs uses and popular tradition. 

We have expressly chosen to focus only on ‘traditional uses’, because our interest concentrated on collecting information about the use of WEPs, leaving aside—for the time being and intentionally—all ‘new’ food uses of plants recently acquired in the wake of new experimentations in the area of gastronomy and nutrition. This choice was also due to the need to contain—as much as possible—the margin for erroneous identification of taxa which, without this a priori selection would most likely have been higher [65].

Attention was thus paid to redundant work, that is to say where the same set of data had been published, by the same authors, in separate contributions for the same geographical area.

Finally, scientific contributions of a methodology, conceptual, demo-anthropological or generic nature have been excluded, where no reference was made to alimurgic species e.g., [6,36,66]. 

In advance of this selection work, a list was compiled with a total of 358 published works (Supplementary Materials Table S2).

Each taxon has been associated with the part used and with the methods through which it is consumed part of food preparations. These categories mainly refer to those listed by [25], with some changes in terms of methods, which have become more articulated in order to provide more detailed information (Table 2).

As regards the parts used, reference was made to the following categories (Table 3), grouped according to the position in the plant and to the function performed:

The use of galls, recorded only for two species (*Rhododendron ferrugineum*, *R. hirsutum*) have not been considered while that of the floral nectar in *Gladiolus italicus*, *G. byzantinus* (=*G. communis*), and *Lamium orvala* has been included in flowers/flower buds in Table 3.

Moreover, for WEPs, the calculation on a national and regional scale included both chorology spectrums based on the chorotypes selected by [67] and biological ones [67,68].

Further processing steps involved titles published for the purpose of highlighting the trend over the past 100 years and their contribution for each administrative region.

## 3. Results

### 3.1. Edible Plants Entered in the Database

The reference to 358 publications on alimurgy-related subjects has allowed us to select 343 papers which led to introducing 1103 taxa in the database. This value corresponds to 13.09% of the 8426 entities chosen for comparison from the checklists of Italian flora [60,62].

The latter have been divided into the following categories: *Pteridophyta* (8 taxa), *Gymnospermae* (13 taxa), *Angiospermae* (1082 taxa, of which 964 *Dicotyledones* and 118 *Monocotyledones*).

**Figure 2:**
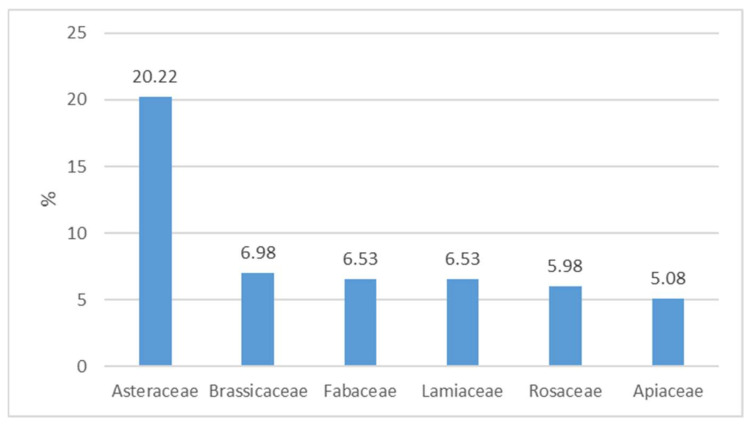
Percentage composition of the main families of Italian Wild Edible Plants (WEPs).

Of the 108 families included, the most widespread appeared to be Asteraceae with 20.22% of taxa, followed by Brassicaceae (6.98%), Fabaceae and Lamiaceae (6.53%), Rosaceae (5.98%), and Apiaceae (5.08%) (Figure 2). These six families account for 51.31% of the total taxa in the database. The prevalence of Asteraceae further confirms the records in numerous publications on the subject of phyto-alimurgy [20].

**Figure 3:**
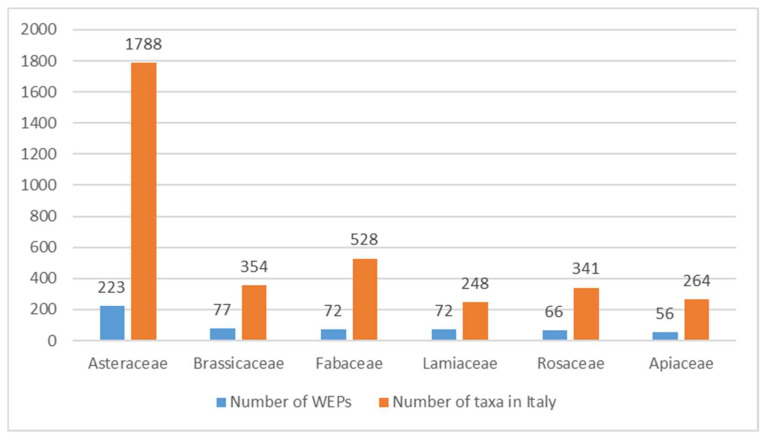
Number of Wild Edible Plants (WEPs) recorded (blue column) and total number of taxa in Italian flora of the most frequent families in the Database AlimurgITA.

The number of WEPs recorded by family was then compared to the taxa present in the same family for Italian flora (Figure 3).

Thirty-seven families (34.26% of the total), are represented in the database by one taxon only.

**Figure 4:**
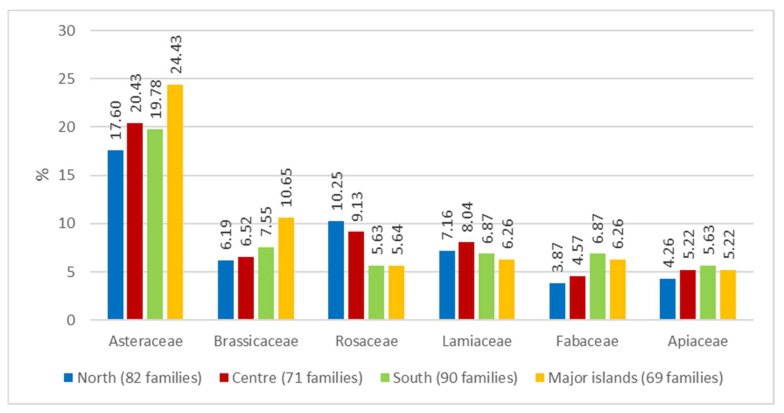
Percentage of the main families of Wild Edible Plants (WEPs) by geographical area.

An assessment was then made of the breakdown of the most frequently found families along the geographical gradient of four geographical areas (North, Centre, South, Major islands) (Figure 4).

Asteraceae and Brassicaceae appear to be more widely used in the islands (24.43% and 10.65% respectively), Rosaceae are dominant in the North and the Centre (10.25% and 9.13% respectively), while the use of Fabaceae is slightly lower in the North and the Centre (3.87% and 4.57% respectively). Apiaceae show a prevalence in the southern sector (5.63%). The gradual increase in the use of Asteraceae in the island area was already recorded earlier in other ethnobotanic contributions [69,70].

There are 494 genera, and those with a number of reported taxa equal to or higher than 10 are *Crepis* (19 taxa), *Allium* (18), *Lathyrus* (16), *Rumex* (15), *Prunus* (13), *Artemisia*, *Brassica, Carlina, Centaurea*, and *Malva* (10); these data confirm the close match between the most representative families and the most frequently recorded genera.

Table 4 includes a list of the 19 taxa most widely used for alimurgic purposes (from 100 records upwards). *Borago officinalis*, *Cichorium intybus*, *Foeniculum vulgare*, *Urtica dioica*, *Taraxacum officinale* (group) s.l., *Sonchus oleraceus Papaver rhoeas* subsp. *Rhoeas*, and *Asparagus acutifolius* are the most frequently recorded species (from 150 records upwards), found in more than 46% of the publications considered, and used in at least 18 Italian regions.

**Figure 5:**
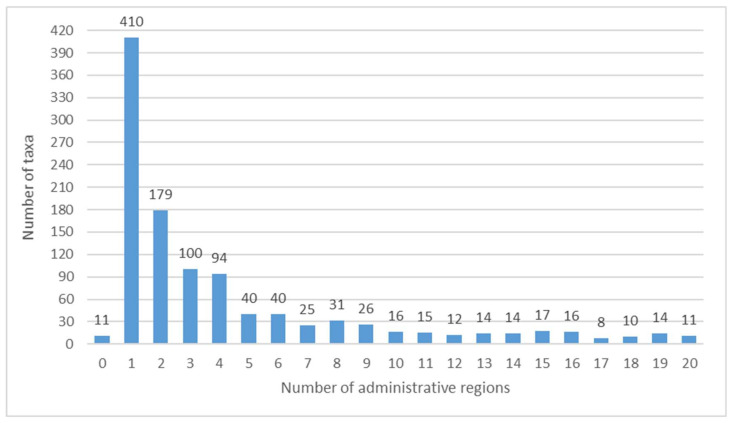
Number of administrative regions where the taxa have been cited.

As many as 234 taxa (21.21%) are recorded only once, and 410 taxa (37.17%) are mentioned only for one region (Figure 5). In the figure the value of 0 is due to the absence for 11 taxa (1.00%), of any specific regional reference, so the information refers to Italy in general. These 11 entities (*Ambrosia artemisiifolia*, *Argentina anserina* subsp. *anserina* (=*Potentilla anserina*)**,** *Apios americana*, *Bassia scoparia*, *Butomus umbellatus*, *Chaerophyllum bulbosum* subsp. *bulbosum*, *Cynomorium coccineum* subsp. *coccineum* (=*Cynomorium coccineum*), *Nelumbo nucifera*, *Nuphar lutea*, *Sagittaria sagittifolia*, *Triglochin maritimum*) all come from [71] (apart from *Apios americana*, also cited by [72], and *Sagittaria sagittifolia*, also cited by [73]) and it is strange that these citations have never been found in any Italian region.

### 3.2. Italian Wild Edible Plants Divided by Region and by Geographical Area

**Figure 6:**
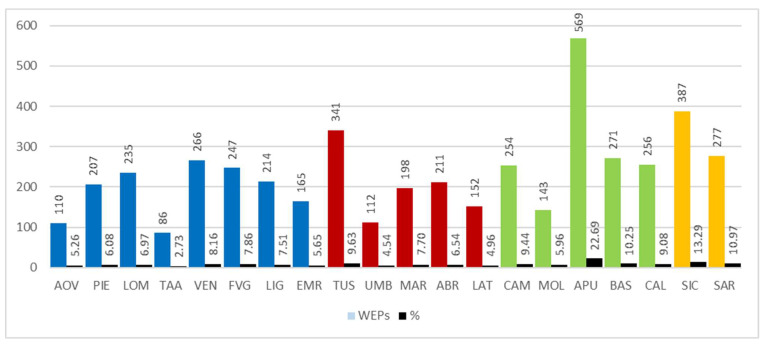
Number of Wild Edible Plants (WEPs) by region in respect of the percentage composition of taxa present in the regional flora. Abbreviations of the administrative regions are shown in Figure 1.

The analysis of results involved calculating the number of edible spontaneous taxa used for each Italian region. It is worth noting (Figure 6) that the region with the largest number of recorded taxa is Apulia (569), followed by Sicily (387) and Tuscany (341). On the other hand, the regions with the lowest use of WEPs are Trentino-Alto Adige, Aosta Valley, Umbria, and Molise with 86, 110, 112, and 143 taxa respectively.

**Figure 7:**
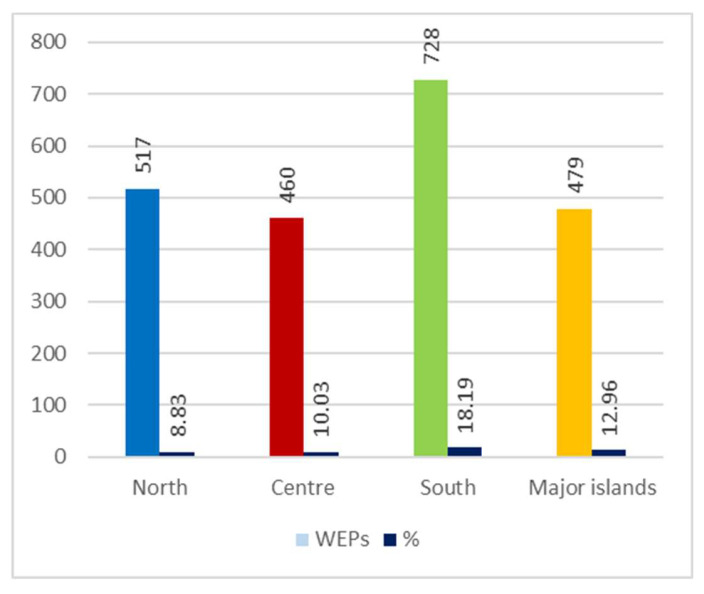
Number of recorded Wild Edible Plants (WEPs) by geographical sector and percentage comparison with regional flora.

Among the four geographical areas considered, the one with the most taxa mentioned (728) is the South, followed by the North (517), Major islands (479), and Centre (460), even though—compared to regional flora—the WEPs percentage is higher to the South then the Major island (Figure 7).

The taxa excluded from the calculation, according to the status defined by [60] (casual alien, absent, doubtfully occurring, reported by mistake, no longer recorded) and updated to date according to [61] are reported, region by region, in Table 5.

### 3.3. Relationship between Edible Plants Recorded by Region and Those Potentially Present

Regional spontaneous edible flora has been linked with the species which could be found in the same areas but which are not used. These latter data have been derived by selecting the alimurgic species found in the region, based on local flora [60,62], from the total recorded in the AlimurgITA Database (1103 taxa).

**Figure 8:**
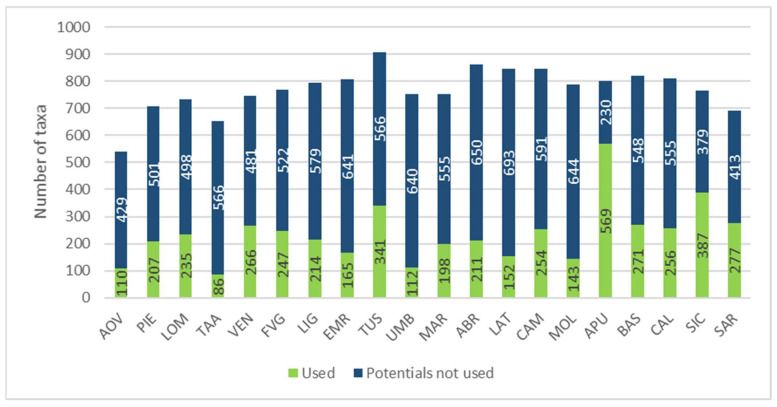
Regional comparison based on the number of recorded and potential alimurgic taxa. Abbreviations of the administrative regions are shown in Figure 1.

Figure 8 shows that WEPs actually account for a small percentage in respect of the potential regional edible flora, ranging from 13.19% (Trentino-Alto Adige) to 71.21% (Apulia). 

**Figure 9:**
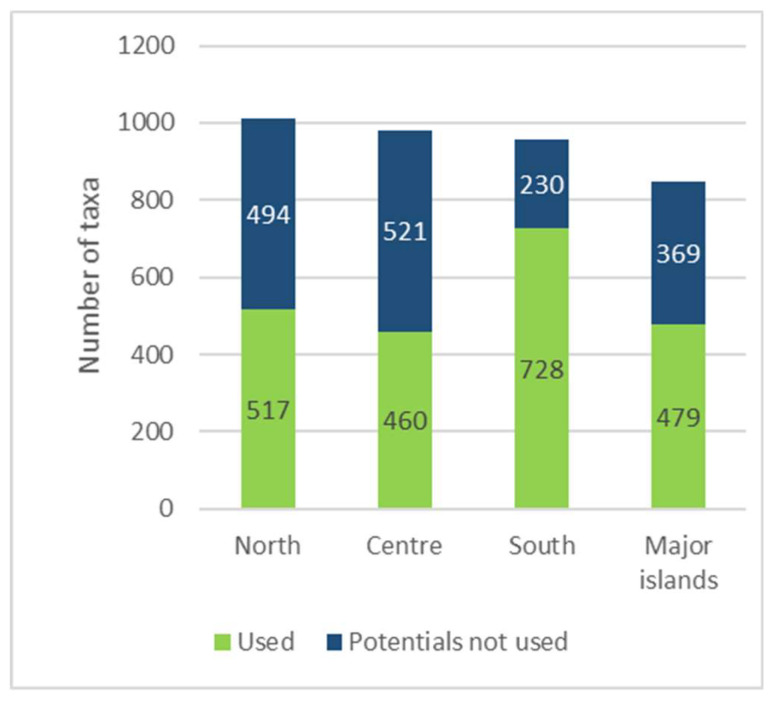
Comparison by geographical area between the number of recorded and potential alimurgic taxa.

In order to provide an overview, the regional data have then been aggregated by geographical area (Figure 9).

This comparison serves the purpose of making assumptions regarding the overall situation of alimurgic knowledge, which in turn can be associated with the level of erosion of traditional knowledge (*TK*) [64], due to socio-economic transformation or to the degree of ethnobotanic exploration, highly variable from region to region.

### 3.4. Parts of WEPs Used

**Figure 10:**
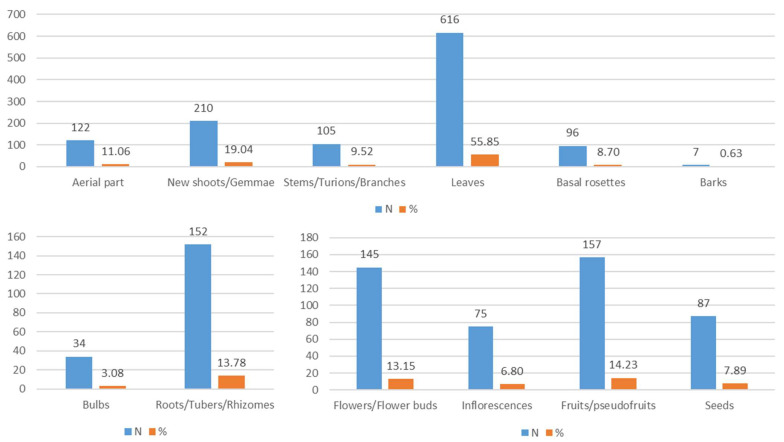
Parts of the plant used and their frequency in the whole database.

In Italy, as regards the most widely used edible parts in the culinary tradition, these include leaves (616 records), new shoots/gemmae (210), roots/tubers/rhizomes (152), fruits/pseudofruits (157), and flowers/flower buds (145) (Figure 10). There are only eight records of resin/sap/latex (0.75%).

### 3.5. Methods for Use of WEPs

**Figure 11:**
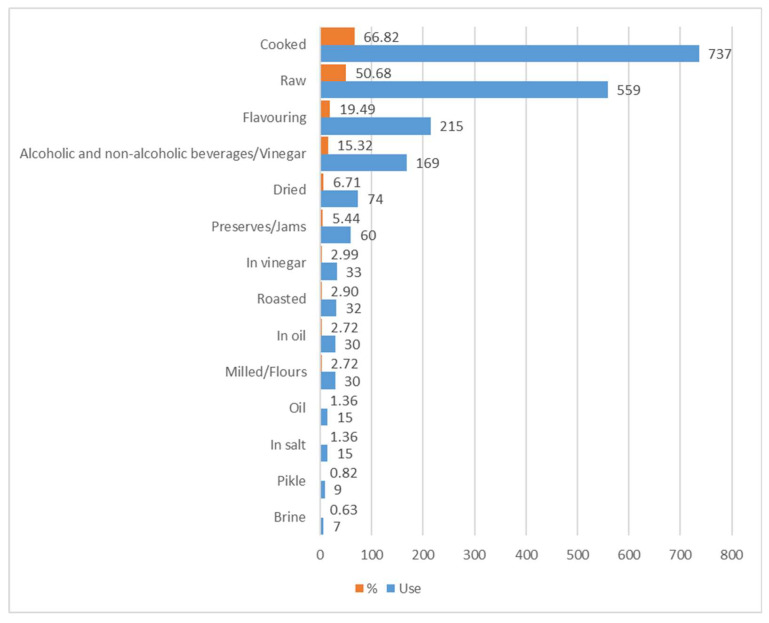
Methods for use of edible species and their frequency in the whole database.

Further analysis has led to processing data related to the number of records for each of the methods of use (Figure 11). This assessment has shown that the most widespread modes of consumption are, in this order, cooked (737 records, amounting to 66.82%), raw (559 records, 50.68%), flavouring (215 records, 19.49%), and beverages/vinegar (169 records, 15.32%).

The cooking of WEPs follows articulated local recipes, and is extremely varied (boiled, stewed, in omelettes, fried, to make vegetable broths, risotto, soups, stuffed pasta, savoury pies, etc.). As regards the most widespread use, it is worth noting that today there are many spontaneous species which are eaten raw in a salad, either on their own or mixed with other herbs. This latter type of use is advantageous compared to the former because there is no loss in terms of nutritional value [74], on the other hand it is potentially risky. It is worth remembering that when food is eaten raw, there is no alteration of any toxic or dangerous substances present. Finally, they are frequently used for beverages (alcoholic and otherwise) derived from fermentation or infusion in water or alcohol, mainly using fruits and flowers as a base, which also become part of local tradition.

Wide and well-documented use is made of edible wild fruit: loquats, raspberries, wild strawberries, blackberries, blackcurrants are eaten fresh, in fruit salads, jams, desserts, biscuits, syrups, and the like. Several species are used to prepare hot beverages such as herb teas (e.g., *Mentha* sp.pl., *Melissa officinalis*).

### 3.6. Potentially Toxic Species 

A fairly large number of taxa, which in literature appear to be consumed as food, can be considered in some way toxic. Many of them are species which—apart from being widespread across the country—are largely used and closely connected to the Italian culinary tradition; they include for example *Borago officinalis* and *Dioscorea communis*; in other instances, they are species whose use as food is really sporadic, and in any case limited to extremely small areas, for example *Polygonatum multiflorum* and *Lonicera etrusca*, with very few records in literature. In all cases they are species which contain several categories of chemical compounds (active ingredients, phyto-complexes) whose toxic effects range from high to bland. An example of these highly toxic species, whose use is clearly documented, is *Conium maculatum*; taken in certain doses the latter is considered deadly due to the presence of the alkaloid coniine, even though in some parts of Sardinia the stems of young plants without the bark are eaten [75]. Among the highly toxic species it is also worth mentioning *Lactuca virosa*, whose young leaves are sometimes eaten as salad [75,76] and *Euphorbia lathyris* whose buds were used in Veneto instead of capers [77].

Species with medium or low toxicity include those which can prove dangerous only in cases of excessive and prolonged consumption, for example those in the genus *Rumex* and *Oxalis* due to the presence of oxalates, or of *Amaranthus*, which can accumulate nitrates. In other species, toxic substances are concentrated only in some organs, possibly absent—or in any case found only in amounts which are not significant—in the parts used. This is the case, for example of *Clematis vitalba* which contains ranuncholine, a glucoside which, by hydrolysis, releases protoanemonine, with a strong irritant and blistering effect [21,25,78,79]; however only the buds of this plant are eaten (where the concentration of ranuncholine is smaller), always cooked (protoanemonine is thermally unstable). Also the type of preparation, therefore, can make a difference: in order to make some bulbs edible, for example *Cyclamen hederifolium*, *C. repandum*, *Bellevalia romana*, prolonged cooking was followed by macerating in cold water [80,81,82] to eliminate any excess of both bitter and toxic substances (saponins, cyclamins, and histamines).

**Figure 12:**
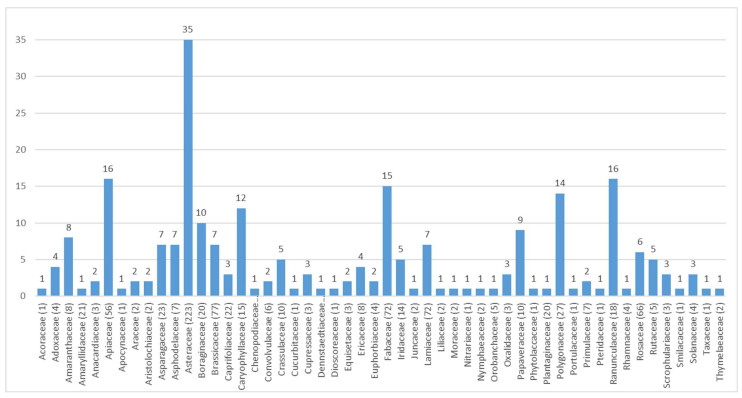
Distribution of toxic taxa of Italian alimurgic flora included in the database AlimurgITA.

As can be seen in Figure 12, it appears that—in some families—most WEPs are toxic in one way or another; in other cases, practically all of them are, for example Boraginaceae such as *Borago officinalis*, *Symphytum officinale*, and *S. tuberosum* which contain unsaturated pyrrolizidine alkaloids [83,84]; if used frequently these plants have a hepatotoxic and blandly mutagenic action [74]; moreover, experiments on animals have shown their carcinogenic action, through a genotoxic mechanism [85].

### 3.7. Biological Spectrum

**Figure 13:**
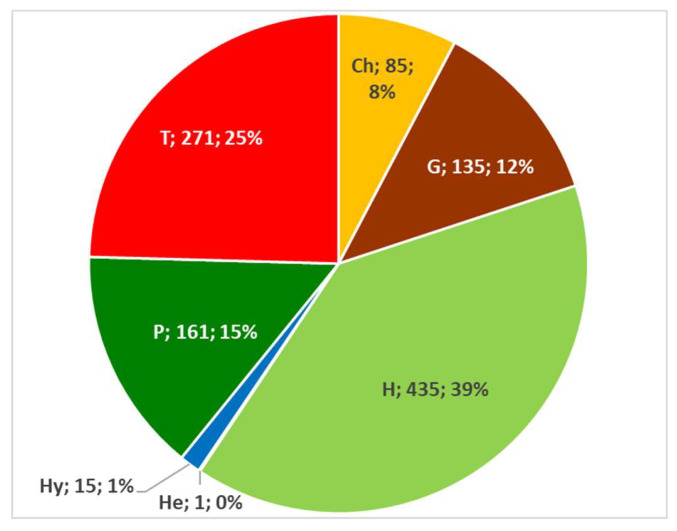
Composition in absolute and percentage value of the Italian edible spontaneous taxa biological spectrum. Ch = Chamaephytes; G = Geophytes; H = Emicryptophytes; He = Helophytes; Hy = Hydrophytes; P = Phanerophytes; and T = Therophytes.

Examining life forms, out of the 1103 taxa recorded, it has been shown that the most widely represented can be divided as follows, in decreasing order: emicryptophytes (39%), therophytes (25%), geophytes (12%), phanerophytes (15%), chamaephytes (8%), hydrophytes (1%), and helophytes (0.09%) (Figure 13). Table S2 of the Supplementary Materials shows a breakdown by subforms.

The widespread presence of Hemicryptophytes is mainly due to their habitus, because they be either biannual (*Crepis vesicaria*, *Daucus carota*) or perennial species (*Cichorium intybus*, *Plantago lanceolata*, *Foeniculum vulgare*). Among the various sub-forms, the most relevant in percentage terms include scapose plants (H scap), accounting for 22.48%, of which mainly the basal rosettes and leaves are used (e.g., *Cichorium intybus*). 

As regards Therophytes, consisting almost exclusively of scapose plants (T scap—22.39%), it is their tender leaves that are generally eaten (*Chenopodium album*, *Portulaca oleracea*, *Borago officinalis*), then possibly their aerial part.

As for Phanerophytes, which are mainly represented by scapose plants (P scap—4.35%), it is their fruits that are mainly used, both fresh and dried (e.g., *Sambucus nigra*, *Juniperus communis*, *Ceratonia siliqua*). The group of phanerophytes and nano-phanerophytes (P and NP), trees and small shrubs, provide fruits and wild seeds; on the other hand, new shoots are mostly collected from liana phanerophytes (P lian).

Geophytes, both bulbous (G bulb—5.80%) and rhizomatous (G rhiz—5.80%) are mainly used for their underground part (tubers, roots, bulbs) (e.g., *Ficaria verna*, *Helianthus tuberosus*, and *Muscari comosum* (=*Leopoldia comosa*)).

The most widely used parts of Chamaephytes, especially aromatic suffruticose plants (CH suffr—3.90%) rich in essential oils used to flavour dishes (e.g., *Thymus vulgaris*, *Satureja montana*) are branches and leaves.

Hydrophytes (*Trapa natans*, *Acorus calamus*, *Glyceria fluitans*, *Posidonia oceanica*), mainly rooting plants (I rad—1.27%), are scarcely represented and not associated with the use of specific parts of the plant. Their consumption is limited to four regions (Lombardy, Veneto, Liguria, Apulia). The only helophyte found (*Phragmites australis*) is used just in the Apulia region; the parts used of the latter are rhizomes, tender leaves and top shoots, either raw in salads and cooked/stewed or in omelettes.

### 3.8. Chorology Spectrum

**Figure 14:**
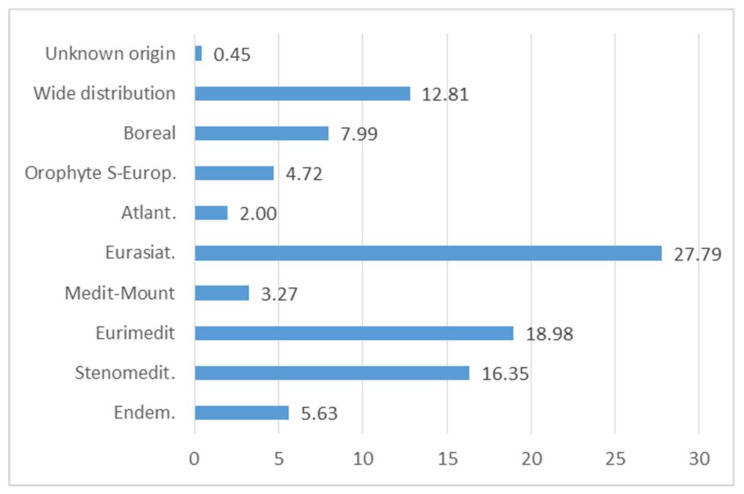
Percentage composition of the chorologic spectrum of Italian of Wild Edible Plants (WEPs).

**Figure 15:**
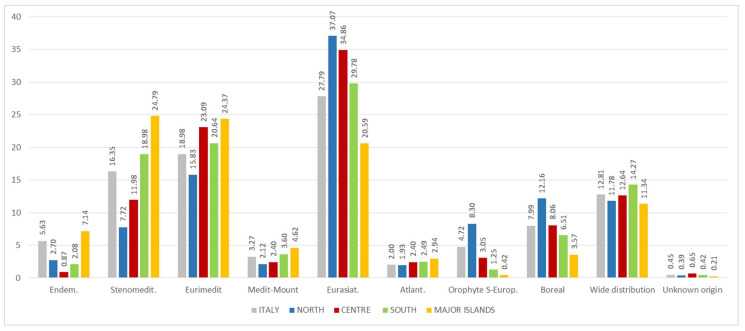
Percentage composition of the chorologic spectrum of Italian edible spontaneous taxa by geographical region.

From a phyto-geographical perspective, it is interesting to note that most of the food flora comes from the Mediterranean region, with a strong presence of Euri-Mediterranean (18.98%) and of Steno-Mediterranean plants (16.35%); this highlights—in the gastronomic field—the connection between the various cultures in the Mediterranean area [15,16,69,70] (Figure 14 and Figure 15). In fact, some plants are used in different parts of Italy and the Mediterranean, for example *Papaver rhoeas* subsp. *rhoeas*, *Sonchus asper*, or *Reichardia picroides* [71,86,87,88,89,90,91].

The high incidence of Eurasian species, about 27.79%, is due to strong bio-geographical connections between the flora contingent in the Italian Apennine region and areas belonging to Eurasia. 

Boreal species connected with Northern Europe or mountain environments account for 7.99% (Euro-Siberian and Circumboreal species). There is a limited amount of both SE-European Orophytes (4.72%) distributed along the mountain strip of Balkan areas, and Atlantic (2.00%) species whose distribution area is focused on the Atlantic coast of Europe.

Only 5.63% of the total alimurgic flora is represented by endemic species, divided in an extremely discontinuous way, with low values in the central sector (0.87%), and on the other hand a strong accentuation in the island area (7.14%).

Further significant data resulting from the chorology analysis are related to species with a wide distribution present in Italy accounting for 12.81%, a value which is fairly similar in the various geographical sectors. Within this chorotype, specific consideration has been given in this case to ‘adventitious’ plants, i.e., alien species, now naturalized, which—over the centuries—have found a phytoalimurgic collocation, fully entitled to fit within the multi-faceted framework of traditional regional cuisines. From an ecology perspective, adventitious plants can be considered in most cases species with a pioneering approach and a marked colonizing activity, often invasive, in synanthropic nitrophyle or ruderal habitats [92]. This set of characteristics has allowed them to establish themselves rapidly also on large surfaces. It therefore seemed appropriate to further divide this chorotype into its sub-categories for the purpose of attempting to historically connect and reconnect, although in an approximated form, the beginning of the use of some species in the Italian culinary tradition. In this regard, Table 6 shows that the main flora supplies of alimurgic species, with the exception of Cosmopolitan and Subcosmopolitan plants, come from the American and Asian continent and from desert areas from the Mediterranean to central Asian region (Mediterraneo-Turanian species).

### 3.9. Bibliography Contributions from 1918 to 2020

**Figure 16:**
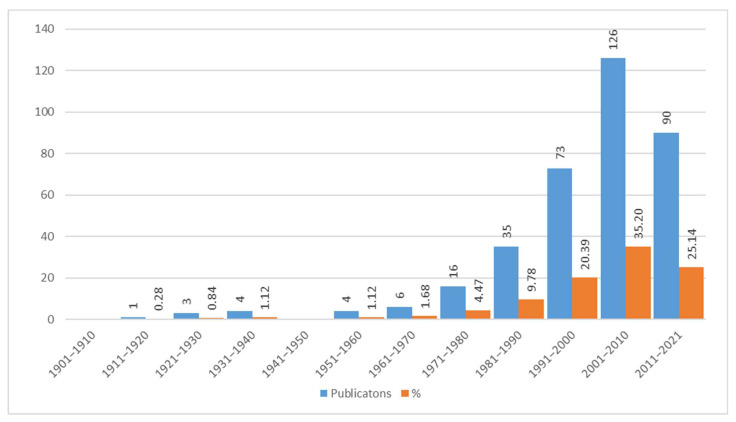
Publications (358) on the alimurgic theme per decade, expressed in absolute and percentage figures.

Considering the state of our knowledge regarding wild edible plants, further analysis has been focused on how this knowledge has become more in-depth over time proportionally to the degree of interest shown in them by the audience of specialists in this discipline. Starting from the first modern publication by [57] to the present day, the overall bibliography trend has been outlined with a 10-year interval division. From Figure 16 it appears clearly that the increase in texts published started to become significant during the 1991–2000 decade, reaching its peak in the next decade, then dropping in the 2011–2020 decade. There have been 289 publications on WEPs (accounting for 80.73% of the total) produced between 1991 and 2021, which is evidence of the growing interest in the subject of alimurgy.

### 3.10. Bibliography Contributions for Each Italian Region

**Figure 17:**
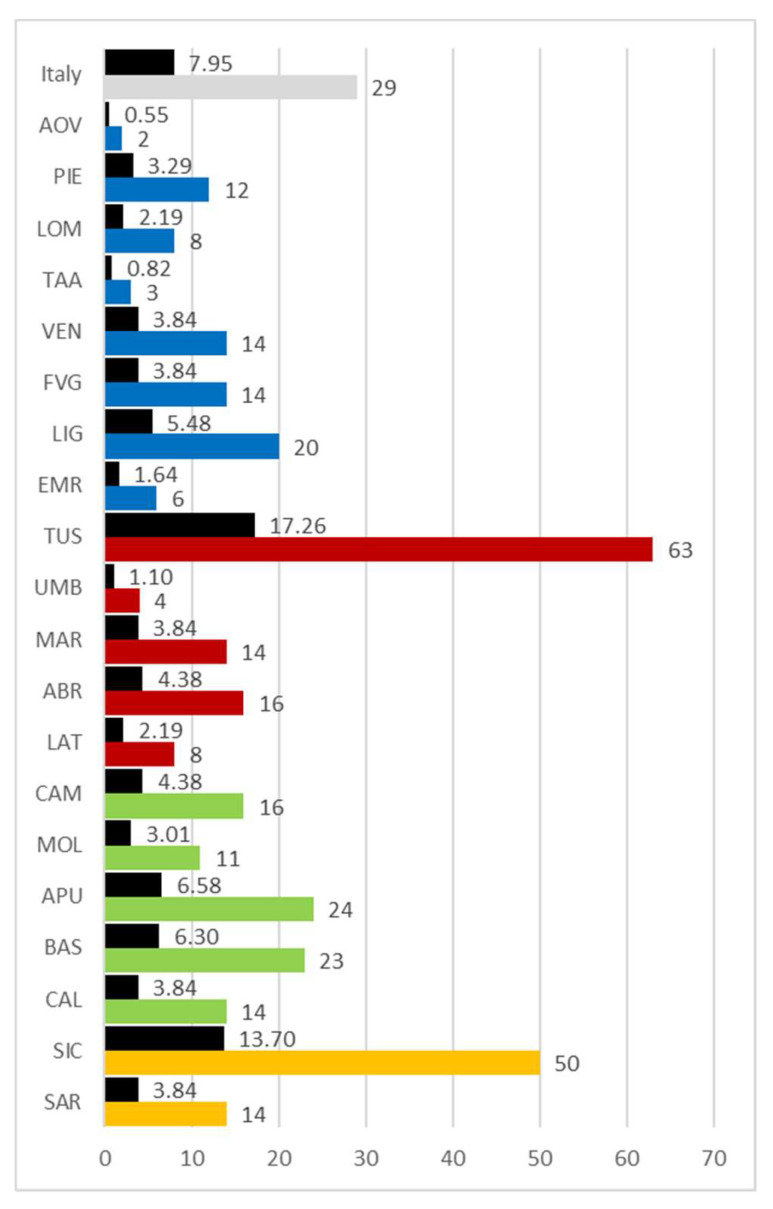
Number of texts published by region or in Italy based on absolute (colored columns) and percentage (black columns) values. Abbreviations of the administrative regions are shown in Figure 1.

Considering the amount of bibliography contributions published for each region, according to the criteria established in the methodology section, the available data show very clearly (Figure 17) that the three regions with the largest number of published specialized texts are Tuscany (63), followed by Sicily (50) and Apulia (24). There are numerous regions with limited bibliography contributions: Aosta Valley (2), Trentino Alto-Adige (3), Umbria (4), and Emilia Romagna (6). Tuscany, with its abundant production, accounts for 17.26% of Italian literature overall, while Sicily accounts for 13.70%.

In total, the taxa which should be considered somehow toxic are 242, accounting for 21.94% of WEPs in Italy. 

In any case, despite the presence of active ingredients potentially harmful for human health, their consumption for centuries [93,94] is evidence that they are essentially harmless if ingested. Of course, the limit between the two categories (toxic/non toxic) is arbitrary, because it is difficult to establish: as always the following concept applies: “All things are poison and nothing is without poison; only the dose makes a thing not a poison” according to the famous sentence by Paracelsus.

## 4. Discussion

The 1103 taxa of edible flora entered in the database AlimurgITA account for 13.09% of Italian flora. There has thus been an increase by 275 taxa compared to the 828 recorded in the database of CET (Centro Etnobotanico Toscano) which also includes several cultivated plants [20]. A further 177 taxa listed in the bibliography consulted, and considered for this project, have been excluded from the total number of alimurgic plants because they are cultivated or considered occasional Adventitious (for a definition, please refer to Materials and Methods).

The high frequency of edible Asteraceae, accounting for 20.22% of total WEPs, is apparently associated with the high number of taxa in Italian flora, to their palatability which is combined with their large availability in several seasons. The basal rosette, the most widely consumed part, is available even during the autumn-winter. It is also worth mentioning the low toxicity of this family, mainly with regard to the subfamily of *Cichorioideae* to which most of the recorded WEPs belong.

The fact that Asteraceae are more representative has also become apparent in a number of studies, therefore it is confirmed to be one of the most widely used in Italy [20,74,95], especially in insular regions of the country [69,70].

The regional distribution of WEPs is far from homogeneous, ranging from a minimum of 86 taxa recorded in Trentino-Alto Adige to the maximum of Apulia, with 569 taxa. The data from Apulia derives mainly from the contribution by Bianco who, in one publication alone [96], reported as many as 564 taxa (of which 156 not reported in previous papers); all this has also affected the distribution of aggregated data by sector in southern Italy, the area with the largest number of taxa. Trentino Alto-Adige, Emilia-Romagna, and Umbria show the lowest numbers of WEPs in Italy.

In some cases (e.g., Veneto, Tuscany, Sicily) the number of taxa mentioned in the database AlimurgITA appears to differ, although just by a few units, from the one recorded in summary publications of a regional nature [70,77,97,98]. The difference is due to the different criterion for choosing WEPs, to the different nomenclature system and to any updates of lists with the acquisition of new data.

**Figure 18:**
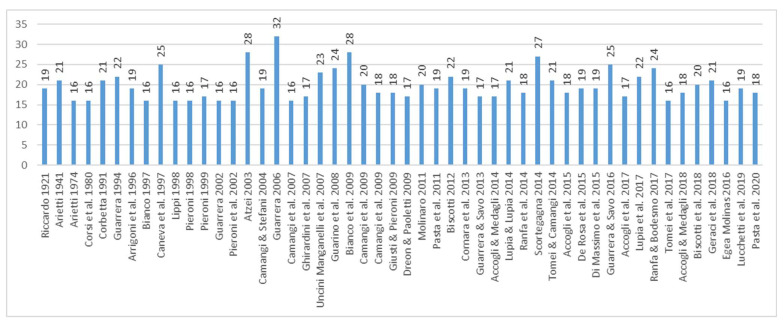
Number of taxa cited by [57] and also reported in publications of subsequent years.

The large number of alimurgic taxa by region can be interpreted in various ways. A first theory is associated with the assessment of the time span during which the alimurgic studies have taken place: the more distant they are from the current time, the higher the probability that it might be possible to have access to cultural heritages which are richer and even more strongly rooted to traditional used. The time span is also related to another element (which is often neglected), namely the continuity of recording within the same territorial framework, which has made it possible to monitor the persistence in the use of some species. On a national scale, for example, the 32 taxa listed in the publication by [57], then entered in the database (*Achillea millefolium*, *Asparagus acutifolius*, *A. officinalis* subsp. *officinalis*, *Bellis perennis*, *Bistorta officinalis* (=*Persicaria bistorta*), *Bunias erucago*, *Campanula rapunculus*, *Capsella bursa-pastoris* subsp. *bursa*-*pastoris*, *Diplotaxis tenuifolia*, *Eruca vesicaria*, *Humulus lupulus*, *Hypochaeris radicata*, *Lapsana communis*, *Loncomelos pyrenaicum* (=*Ornithogalum pyrenaicum*), *Melissa officinalis*, *Nasturtium officinale*, *Papaver rhoeas* subsp. *rhoeas*, *Parietaria officinalis*, *Plantago lanceolata*, *P. media*, *Portulaca oleracea*, *Poterium sanguisorba* (=*Sanguisorba minor*), *Salvia rosmarinus* (=*Rosmarinus officinalis*), *Sinapis arvensis* subsp. *arvensis*, *Sisymbrium officinale*, *Sonchus arvensis*, *S. asper*, *S. oleraceus*, *Symphytum tuberosum*, *Taraxacum officinale* group, *Tragopogon pratensis*, *Urtica dioica*), have been mentioned in various ways over time by several authors and in publications both as bibliographic compendium and territorial ethnobotanic census (Figure 18).

Among these taxa, those which are no longer mentioned include Loncomelos pyrenaicum (=Ornithogalum pyrenaicum), since 2017, Plantago media and Bistorta officinalis (=Persicaria bistorta), since 2018, Lapsana communis, Sisymbrium officinale, and Symphytum tuberosum subsp. angustifolium since 2019.

A second theory is related to floral and coenological diversity, which in turn is associated with the geographical extension and complexity of the regions surveyed, large sections of which may not be explored in the absence of regional overviews.

There is a third theory related to the latter, connected with the number of publications, even though this element cannot always be correlated to the degree of knowledge. For example, Tuscany, the region which provided the largest number of publications on the subject of alimurgy (sometimes redundant in terms of information) shows similar levels of knowledge compared to Veneto, Calabria, and Sardinia, characterized by few but substantial synthesis works [16,75,77,99].

A last theory refers to the use of the territory and to its impact in terms of erosion in traditional culture. The limited number of WEPs in Emilia-Romagna, for example, could also be due to agricultural practices on an industrial scale which—over the past few decades—have radically changed the farming society, leading to a rapid reduction of ethnobotanic knowledge (and therefore of WEPs).

The remarkable differences in the number of species recorded from region to region can thus be associated also with the level of erosion in the transmission of culture. Attempting to estimate, even hypothetically, the entity of this loss, was one of the objectives of this project. 

This is why we calculated the ratio between edible plants recorded by region and the species potentially present.

The results, in percentage terms on a national scale, have shown a use of alimurgic flora accounting for 7.6% compared to the potential value; as regards regional flora, WEPs account for 2.73% in Trentino Alto-Adige and 22.69% in Apulia. As for southern regions and major islands, the values remain high, which is evidence of on-going and capillary ethnobotanic explorations, complemented by traditional culture legacies which are still present, vital, and can therefore be detected. In this area there are some specific cultural provisions of ancient races, which are also important in order to establish the ethnic-historical connection, which sometimes still exists, with eastern Adriatic countries (Greece, Albania, Croatia) [80,100,101,102,103,104]. An example of this is the influence of Greek communities in Calabria, developed as a consequence of the historical migrations which occurred in Greece. More than half of the plant species from the Graecanic area (58%) are also used as edible plants in Greece [105,106] and there are some species (*Chrysanthemum segetum*, *Hedypnois cretica*, and *Lotus edulis*) commonly used in Greece and in the Graecanic area, but not in other parts of Italy [107].

It is finally worth noting the substantial contribution of publications from the past 20 years for the southern and insular areas as further evidence of the persistent vitality of cultures related to farming and shepherding.

Their availability for most of the year, their palatable taste, territorial frequency, specific biomass, ease of recognition and therapeutic virtues are all factors which—taken together—lead to the success in the use of a given species. This is the case of *Borago officinalis*, *Urtica dioica*, *Sonchus oleraceus*, *Taraxacum officinale* group, *Papaver rhoeas* subsp. *rhoeas* which are widely eaten and present in all recipe books of traditional cuisine in every Italian region, as evidence of still existing ethnobotanic connections. 

Nevertheless, some differences have been detected in the gathering and consumption of wild plants between the various Italian regions. In terms of families recorded, in the North and Centre there is a prevalence of Rosaceae, in the South of Brassicaceae and on the islands of Asteraceae. These data match what was in part recorded by [20,64].

As a matter of fact, the use of each species on the Italian territory is related not just to its area distribution but also to frequency, that is to say its availability in the region, a factor which would explain the anomalies in the failed reporting of its use. Species which are widely used in Italy—such as *Clematis vitalba* and *Asparagus acutifolius*, for example—do not appear to be used in Aosta Valley and in Trentino-Alto Adige due to their rarity, even though they are present in regional flora [60,62]. Nevertheless, other taxa show anomalies related to discontinuity in distribution with geographical sector leaps which are difficult to interpret. Reference can be made, for example to the absence of *Sambucus nigra* in Umbria, of *Laurus nobilis* in Aosta Valley and Umbria, of *Rosa canina* in Umbria alone, of *Plantago major* in Latium *Clinopodium nepeta* subsp. *nepeta* in most regions in the north, and of *Hypochaeris radicata* which shows gaps in every region.

The current lack of reference to the use of a given plant in a specific region could be due to its limited availability, to the decline in its use of time, to its absence in tradition, or simply to the fact that it has not been intercepted by ethnobotanic studies. At the moment we believe that it is not possible to establish which of these theories (ecological and cultural) is more sensible.

It is also worth mentioning that the most accentuated and deeply-rooted preference of taste in some regions, for example for the bitter flavour, which is more appreciated in southern Italy than in the North [64,102,103].

Generally speaking, when talking of ‘traditional’ use of a given species, collective imagination places it many centuries before the modern age. This is not always true because the use of some species (and of recipes) which are undisputedly considered as belonging to the cultural heritage of one area, may date back just one or two centuries.

In some circumstances, the beginning of the use of WEPs can be placed within a specific time frame: this is the case of adventitious species from the New World which have gained ground and later become widespread in Italy thanks to their presence in seeds of other crops (e.g., tomato, potato, corn).

Nor can it be ruled out that some of these adventitious plants might have been grown on purpose to be abandoned later if they should no longer prove convenient or palatable, as for example in the case of *Lepidium sativum* and *Satureja hortensis*.

Specific attention should be paid to endemic or rare species. Excessive gathering of these species for edible use could lead to contraction phenomenon in populations, with risks in terms of preservation in nature of the latter. The precautionary principle also applies when there are two species which are morphologically very similar, one of which rare and the other frequent which can be mistaken one from the other, leading to the disappearance or reduction of population of the rarest species. This is the case of several endemic regional plants which are found and eaten only in one Region, such as *Anchusa capellii* (=*Anchusa crispa*) in Sardinia [75] and *Urtica rupestris* in the Sicily [108] whose use is questioned by [98].

It would therefore be appropriate if, for this category of alimurgic species (or of medicinal species), authors could mention the appropriateness of gathering them in moderation, making reference—if applicable—to regional or national regulations.

The most extensively collected parts of WEPs are leaves, especially if joined into basal rosettes (H ros) which, being easy to collect, guarantee, in rapid times, the acquisition of a satisfactory amount of biomass. Moreover, these parts are tender, very palatable, and versatile in their use. From a nutritional perspective, the fact that leaves are the most widely used part in almost all species should be attributed to their ability to collect fundamental substances, such as mineral salts and vitamins.

More limited use is made of new shoots, gemmae, and flower buds because they are related to very short phenology periods (e.g., *Asparagus* sp. pl., *Capparis* sp. pl, etc.).

The underground systems (roots, bulbs and rhizomes) have a variety of uses because they can be used either as flavouring (gentian, horseradish, liquorice, etc.), or as nutrient reserves (first and foremost starch), given that they can be kept for a relatively long period of time (e.g., garlic, potatoes, onions).

Such a widespread use of fruits, due to their high palatability, is attributable in part to nutritional features: rich in sugar and calories, they are eaten also during famine periods in order to alleviate hunger. The high number of records, in any case, is related also the diversified use which was made of them; their fruits were eaten not only fresh, they were also dried or used to flavour wines and liqueurs.

As regards flowers, their use is mainly associated with preparing miscellaneous beverages and liqueurs. Within the current culinary framework, flowers—due to their pleasant appearance—are mainly used for garnishing dishes.

Even though raw consumption is the easiest and most straightforward, cooking appears to the most widespread because it makes food more digestible, reduces the pathogenic load and allows for eliminating unwelcome characteristics (e.g., toxicity). It is also worth mentioning that, in some cases, cooking enhances the sensorial properties of herbs, mitigating any excessively bitter characteristics. On the other hand, thermal treatments may lead to a series of modifications involving consistency, color, and texture of plant tissues—associated with phenomena related to thermal degradation, oxidation, and leaching—with a subsequent loss of nutrients and anti-oxidant compounds [109,110]. The method of use, also related to preservation and thus to deferred consumption of species over time, also involves complex and multiple preparations (e.g., roasting as pre-requisite for preparing flours or beverages).

## 5. Conclusions

The gastronomic geography of Italy is rich in culinary variants compliant with the morphology of its territories, the climatology of physical environments, the structuring of production activities and the series of historical-cultural and socio-demographic events.

This is the setting for the rich floral diversity of the WEPs used in the national gastronomy tradition.

Over the last century, however, great social upheavals—especially in Europe—have led to a simultaneous trend towards urbanization and depopulation of our inland areas, which are considered the main causes for the disappearance of most of the traditional knowledge and practice as regards wild edible plants [111].

The use of WEPs still thrives, mainly in several small communities, the last strongholds of our farming and shepherding heritage, also thanks to the increased opportunities of being in touch on an on-going basis with the natural environment, compared to urban dwellers. 

The results of this work have shown that the regions where WEPs are most widely used are those where there is still a strong territorial connection which encourages the population and researchers to keep knowledge and skills alive. This phenomenon, with the exception of Tuscany, seems to be concentrated in southern Italian regions (Apulia) and on the islands (Sicily and Sardinia) [70,75,96]. On the contrary, in other Italian regions, the diversity of alimurgic flora shows the lack of knowledge as a whole, considering its extraordinary basic potential.

In this regard, our study can be expected to encourage the in-depth study of alimurgy and more in general of ethnobotany in Italian territories where knowledge appears to be insufficient, not to say scarce.

The wealth and diversification of uses is also due to the impacts of cultures from several ethnic groups, often limited to community minorities found mainly in southern regions and in Sicily (e.g., Slava language communities in Molise, Albanian communities in Molise, Lucania and Calabria, as well as Graecanic communities in Apulia and Calabria) which have created specificities [80,100,101,102,103,104,105,106].

The long list of WEPs in this work, also related to the high floral diversity in Italy, includes all taxa recorded to date in Italian tradition. Unfortunately, it is not possible to establish how many of these taxa are still used or how many are no longer eaten, and therefore should be considered mere ‘historical’ testimonies. 

The gradual erosion of traditional knowledge of WEPs, especially in the last generations [64], is counterbalanced by a widespread phenomenon related to the “craving for nature” and “neo-rurality” which have become something more than a passing trend of our time. Evidence of this is the thriving of numerous texts and recipe books which, over the past 20 years, have been published based on the local tradition and experimenting with alternative uses thanks to a gastronomy culture which is very lively and strongly felt in Italy.

What is the significance of alimurgic plants today?

It is first of all a way of keeping alive a millenary tradition which will be able to survive, as long as it remains functional, without being limited to mere folklore or museum exhibitions. Even though WEPs in Italy are not a staple food, they are often a key element in cultural representations, in identification processes of a community. An example of this is the cross-border identity which is founded on the phrase ‘Mediterranean diet’, even though it appears to be highly diversified in terms of local recipe books. The use of WEPs, to sum up, implicitly means accepting as part of one’s diet the tenets of a traditional model to be followed which is consistent with the characteristics of the territories. The large variety of plant products available spontaneously (complementary to those from the fields and from the orchard), apart from meeting taste requirements is an important nutraceutical product which can prove advantageous in terms of psycho-physical well-being.

In conclusion, studying the socio-psychological components of food, ref. [112] highlights and sums up the above with an insightful sentence: “Whether we are aware of it or not, when we eat we are ingesting not just a specific food, but also the concept (i.e., the culture and territory) associated with it”.

The use of WEPs, furthermore, is also a way of reformulating some paradigms related to production in the farming world. Most of the current agricultural production systems cause environmental pollution because large amounts of fertilizers, pesticides, and herbicides are used. The profound alteration of habitats caused by agriculture is considered the main cause of the loss of biodiversity [13,113,114] with an impact also in terms of reduced connectivity in the landscape. 

The expected human population growth to nine billion by 2050 will require an even more significant increase in agricultural production.

Over the next few decades, the epochal challenge will be to maintain these productive rhythms in the face of biotic and abiotic threats attributed to climate change [33,34,35]. Within this forthcoming scenario, CWRs and WEPs, which are closely linked, can provide a significant and growing contribution to the security of food supplies. Given that they have not crossed the domestication bottleneck [115], WEPs and CWRs maintain a high inherent genetic diversity which allows for their effective adaptation to a wide variety of habitats [116].

WEPs are still a largely unexplored area for farming with a low energy input (including water consumption), as well as for an extension of the availability for humanity of food, vitamins, and nutrients from sustainable sources [117], also including preparations based on herbs, traditional medicine formulas, or new bio-drugs [118,119]. 

Therefore, an overall knowledge of WEPs and of their traditional use in a large territory such as Italy, offers far-reaching prospects in terms of domesticating wild species whose use has already been ‘tested’ by the relevant populations, encouraging their subsequent introduction into production and consumption systems, for example creating demand for species and regulating their gathering in nature, or possibly encouraging their cultivation. 

As rightly pointed out by [120], the COVID-19 crisis has dramatically highlighted the frailty of a trade system based on the globalization of procurement and distribution of food products. The pandemic is leading us towards a new paradigm of the food system consisting of short food chains, or even self-sufficiency. Domestic and community vegetable patches, the traditional agro-ecosystems and farmers’ markets are shaping up as increasingly essential services [118,121].

Within this socio-economic framework, WEPs could constitute a crucial element, as a model of salutogenesis: systemically generating health both in those who practice it and in the environment.

## Figures and Tables

**Table 1:** Partial list of relevant WEP databases

Title	Level	Link	Topic	Language
Acta Plantarum—Flora delle Regioni italiane	Italy	https://www.actaplantarum.org/	A database of the Italian flora, with information on properties and uses of plants	Italian
Cuisine sauvage	Belgium	https://cuisinesauvage.org/les-plantes/voir/	A database of wild edible plants	French
Dr. Duke’s Phytochemical and Ethnobotanical Databases	United State of America	https://phytochem.nal.usda.gov/phytochem/search	A database of wild useful plants, with information on their chemical activities	English
Edible Wild Food	Canada	https://www.ediblewildfood.com/	A blog database on wild edible plants	English
Food Plants International	World	https://foodplantsinternational.com/ (database at: https://fms.cmsvr.com/fmi/webd/Food_Plants_World)	A database of edible plants, with information on their nutritional value	English
Foraging: what to look out for each month	United Kingdom	https://www.woodlandtrust.org.uk/visiting-woods/things-to-do/foraging/	A month-by-month guide to sustainable foraging, what is in season and how to eat it	English
Génial Végétal	France	https://www.genialvegetal.net/	A database of wild edible plants	French
GenResIS (Genetic Reserve Information System)	Europe	http://www.agrobiodiversidad.org/aegro/	Information on recommended locations suited for the establishment of genetic reserves for Avena, Beta, Brassica, and Prunus across Europe	English
Native American Ethnobotany	North America	http://naeb.brit.org/	A database of foods, drugs, dyes and fibers of Native American peoples, derived from plants	English
PHYTOALIMURGIA Piante selvatiche commestibili		https://phytoalimurgia.it/	Database of wild edible plants	Italian, English
Piante innovative	Italy	https://www.pianteinnovative.it/	Information on wild edible plants, with information on the edibility of plants	Italian
Pl@ntUse	Europe	https://uses.plantnet-project.org/en/Main_Page	An ongoing collaborative space for the exchange of information on useful plants	English, French
Plantas silvestres comestibles	Spain	https://www.vivelanaturaleza.com/manual-de-supervivencia/plantas-comestibles/	A blog with information on wild edible plants	Spanish
Plants for A Future	World	http://www.pfaf.org/user/Default.aspx	A database of edible and otherwise useful plants	English

**Table 2:** Mode of consumption

Mode of Consumption	Description
Raw	Plant generally used raw to make salads
Cooked	Plant cooked to make soups, broths, stuffed pasta, etc.
In oil	Plant preserved in olive oil to be used later
In salt	Plant preserved in salt to be used later
In vinegar	Plant kept in vinegar (usually made from wine) to be used later
Brine	Plant kept in brine to be used later
Pickle	Plant macerated to make pickles or sauces
Roasted	Plant which is roasted to make coffee surrogates
Dried	Plant which is dried to be used later
Preserves/Jams	Plant used for confectionery products (jams, preserves, jellies, sweets, etc.)
Alcoholic/non-alcoholic beverages/Vinegar	Plant used to make alcoholic beverages through fermentation, as flavouring for alcoholic beverages (e.g., grappa, wine, rosolio), non-alcoholic beverages (e.g., syrups), or vinegar
Oil	Plant used for food oil extraction
Milled/Flours	This refers to a plant (mainly tubers, rhizomes and seeds in this case) which was boiled then dried, or it was simply dried, then it was milled and finally added to cereal flours
Flavouring	Plant used in small amounts as seasoning for cooked dishes and cheeses

**Table 3:** Function, position, and parts of the plants used

Function	Position	Part
Vegetative	Hypogeal	Roots/tubers/rhizomes
		Bulbs
	Epigeal	Stem/turion/branches
		Bark
		Aerial part
		Leaves
		Young shoots/gemmas
		Basal rosette
Reproductive		Inflorescences
		Flowers/flower buds
		Fruits/pseudofruits
		Seeds
Other		Resin/sap/latex

**Table 4:** List of spontaneous species most widely used for edible purposes

Taxon	N	%	Reg.
*Borago officinalis*	204	62.39	20
*Cichorium intybus*	204	62.39	19
*Foeniculum vulgare*	193	59.02	19
*Urtica dioica*	174	53.21	20
*Taraxacum officinale*	171	52.29	20
*Sonchus oleraceus*	168	51.38	20
*Papaver rhoeas* subsp. *rhoeas*	158	48.32	20
*Asparagus acutifolius*	151	46.18	18
*Clematis vitalba*	145	44.34	18
*Laurus nobilis*	133	40.67	16
*Reichardia picroides*	129	39.45	15
*Silene vulgaris*	126	38.53	20
*Portulaca oleracea*	126	38.53	19
*Sambucus nigra*	123	37.61	19
*Nasturtium officinale*	122	37.31	20
*Malva sylvestris*	117	35.78	20
*Ruscus aculeatus*	108	33.03	17
*Rubus ulmifolius*	106	32.42	19
*Helminthotheca echioides*	105	32.11	15

N = number of records, % = ratio between number of records and reference texts, Reg. = number of administrative Regions where the species is recorded.

**Table 5:** Number of taxa excluded from the calculation of WEPs and reason for exclusion

Reason for Exclusion	AOV	PIE	LOM	TAA	VEN	FVG	LIG	EMR	TUS	UMB	MAR	ABR	LAT	CAM	MOL	PUG	BAS	CAL	SIC	SAR
Casual alien/archaeophyte	2	13	12	3	38	14	16	14	39	2	14	9	12	15	11	9	23	15	11	34
Absent	7	9	1	2	17	9	17	6	12	1	5	2	4	5	9	11	20	19	15	16
Reported by mistake	.	1	.	.	2	2	3	2	2	2	.	2	.	2	.	4	3	3	3	2
Doubtfully occurring	.	.	.	1	1	.	.	.	3	2	.	1	.	1	.	8	4	3	2	.
No longer recorded	.	1	.	.	1	1	.	.	.	.	.	.	.	1	.	9	.	2	1	.
Total number of species	8	24	13	6	59	26	36	22	56	7	19	14	16	24	20	41	50	42	32	52

**Table 6:** Chorology spectrum of wide distribution species, divided by chorotypes

Chorotype	N	%
Africa	3	2.13
America	17	12.06
Asia	17	12.06
Oceania	2	1.42
Adventitia	4	2.84
Cosmopolitan	19	13.48
Mediterraneo-turanian	28	19.86
Paleosubtropical	2	1.42
Paleotropical	4	2.84
Pantropical	2	1.42
Saharo-sindic	2	1.42
Subcosmopolite	38	26.95
Subtropical	3	2.13

## Data Availability

The data will be available when the AlimurgITA website is published

## Supplementary Materials

The following are available online at https://www.mdpi.com/article/10.3390/plants10040743/s1, Table S1: List of taxa excluded from the AlimurgITA database region by region: A = Absent; CA = Casual alien/archaeophyte; NR = No longer recorded; D = Doubtfully occurring; RM = Reported by mistake; P = Present. The cultivated species are marked with an asterisk; Table S2: List of the 358 selected texts; Table S3: Biological Spectrum of subforms.
