# Supplementary material for: Design a Database of Italian Vascular Alimurgic Flora (AlimurgITA): Preliminary Results
Source: Plants (Basel). 2021 Apr 10;10(4):743. doi: 10.3390/plants10040743 (PMC8069721; doi:10.3390/plants10040743)
Supplement: Supplementary file 1 [file plants-10-00743-s001.zip › Paura et al_Database alimurgic flora Italy 2021_Supplementary Materials_Table S3.pdf]

Supplementary Materials Table S3. Biological Spectrum of subforms.

LIFE FORMS	SUBFORMS	N	%
Ch	Ch frut	18	1.63
	Ch rept	12	1.09
	Ch succ	8	0.73
	Ch suffr	47	4.26
G	G bulb	64	5.80
	G rad	3	0.27
	G rhiz	68	6.17
H	H bienn	83	7.52
	H caesp	13	1.18
	H rept	7	0.63
	H ros	66	5.98
	H scand	5	0.45
	H scap	261	23.66
He	He	1	0.09
Hy	Hy nat	1	0.09
	Hy rad	14	1.27
P	NP	38	3.45
	P caesp	65	5.89
	P lian	9	0.82
	P scap	48	4.35
	P succ	1	0.09
T	T caesp	1	0.09
	T par	4	0.36
	T rept	9	0.82
	T ros	2	0.18
	T scap	255	23.12
