# Supplementary material for: Design a Database of Italian Vascular Alimurgic Flora (AlimurgITA): Preliminary Results
Source: Plants (Basel). 2021 Apr 10;10(4):743. doi: 10.3390/plants10040743 (PMC8069721; doi:10.3390/plants10040743)
Supplement: Supplementary file 1 [file plants-10-00743-s001.zip › Paura et al_Database alimurgic flora Italy 2021_Supplementary Materials_Table S1.pdf]

Supplementary Materials Table S1. List of taxa excluded from the AlimurgITA database region by region: A= Absent; CA=Casual alien/archaeophyte; NR= No longer recorded; D= Doubtfully occurring; RM= Reported by mistake; P=Present. The cultivated species are marked with an asterisk

ENTITY	AOV	PIE	LOM	TAA	VEN	FVG	LIG	EMR	TUS	UMB	MAR	ABR	LAT	CAM	MOL	APU	BAS	CAL	SIC	SAR	ITALY
Abelmoscus esculentus (L.) Moench	.	.	.	.	.	.	.	.	.	.	.	.	.	.	.	.	.	.	.	A	.
Allium ampeloprasum L.*	.	RM	.	.	RM	RM	RM	.	RM	.	.	.	.	RM	.	CA	RM	RM	CA	RM	.
Allium ascalonicum Hort.*	.	.	.	.	A	RM	.	.	.	.	.	.	.	.	.	A	.	.	.	.	.
Allium cepa L.*	.	CA	.	.	CA	CA	A	.	CA	.	.	CA	CA	CA	CA	.	CA	A	.	CA	.
Allium porrum L.* (=*Allium ampeloprasum* L.)	.	CA	.	.	CA	CA	A	.	CA	.	.	.	.	.	.	.	CA	CA	.	A	.
Allium sativum L.*	.	CA	CA	CA	CA	CA	A	.	CA	.	CA	CA	CA	CA	A	.	CA	.	CA	CA	.
Aloysia citrodora Paláu	A	A	.	.	A	CA	A	A	CA	.	.	.	CA	A	.	.	A	.	.	CA	.
Ambrosia maritima L. (= *Ambrosia polystachya* DC.)	.	.	.	.	.	.	.	.	.	.	.	.	.	.	.	NR	.	.	.	**P**	.
Anacamptis palustris (Jacq.) R.M.Bateman, Pridgeon & M.W.Chase	.	.	.	.	.	.	.	.	.	.	.	.	.	.	.	.	.	.	NR	.	.
Anethum graveolens L.*	.	.	.	.	CA	.	.	.	.	.	.	.	.	.	.	.	A	A	.	CA	.
Artemisia abrotanum L. (= *Artemisia alba* Turra)	.	.	.	.	.	NR	.	.	.	.	.	.	.	.	.	.	.	.	.	.	.
Artemisia dracunculus L.*	.	.	.	.	A	.	.	.	.	.	.	.	.	.	.	.	.	.	.	A	.
Artemisia glacialis L.	.	.	.	.	.	.	RM	.	.	.	.	.	.	.	.	.	.	.	.	.	.
Avena sativa L.*	.	CA	.	.	CA	.	CA	.	CA	.	.	CA	.	.	.	CA	CA	.	.	.	.
Blitum bonus-henricus (L.) Rchb. (=*Chenopodium bonus-henricus* L.)																NR					
Camelina sativa (L.) Crantz	.	.	.	.	**D**	.	.	.	.	.	.	.	.	.	.	A	.	CA	.	.	.
Camellia sinensis (L.) Kuntze *	.	.	.	.	.	.	.	A	.	.	.	.	.	.	.	.	.	.	.	A	.
Canna indica L.	.	.	.	.	.	.	.	.	.	.	.	.	.	.	.	.	.	CA	.	.	.
Capsicum annuum L.*	.	.	.	.	A	.	.	.	A	.	.	.	.	CA	A	.	CA	CA	.	.	.
Capsicum frutescens L.*	.	.	.	.	.	.	.	.	.	.	.	.	A	.	A	.	.	.	.	.	.
Carlina sicula Ten.	.	.	.	.	.	.	.	.	.	.	.	.	.	.	.	RM	.	.	**P**	.	.
Carthamus tinctorius L.											A										
Centaurea benedicta (L.) L.	.	.	.	.	.	.	.	.	.	.	.	.	.	.	.	.	A	.	.	.	.
Chaerophyllum aromaticum L.								CA	A												
Chamaemelum nobile (L.) All.	.	.	.	.	.	.	.	.	.	.	.	.	.	.	.	.	.	.	.	A	.
Chrysosplenium dubium J.Gay ex Ser.	.	.	.	.	.	.	.	.	.	.	.	.	.	.	.	A	.	.	.	.	.
Cicer arietinum L.*	.	.	.	.	CA	.	.	CA	CA	.	.	.	.	.	A	.	A	A	.	CA	.
Citrullus lanatus (Thunb.) Matsum. & Nakai subsp. *lanatus* *	.	.	.	.	CA	.	.	.	.	.	.	.	.	.	.	.	.	.	.	.	.
Citrus × aurantium L.*	.	.	.	.	.	.	A	.	A	.	CA	.	A	.	.	A	A	A	A	CA	.
Citrus deliciosa Ten.* (= *Citrus reticulata* Blanco)	.	.	.	.	.	.	.	.	A	.	.	.	.	.	.	.	.	.	.	CA	.
Citrus grandis L.* (=*Citrus maxima* (Burm.) Merr.)	.	.	.	.	.	.	.	.	A	.	.	.	.	.	.	.	.	.	.	.	.
Citrus limon (L.) Burm. F.*	.	.	.	.	A	A	A	.	A	.	A	A	A	CA	.	A	A	A	A	CA	.
Citrus medica L.*	.	.	.	.	.	A	.	.	.	.	.	.	.	.	.	.	.	.	.	A	.
Corydalis solida (L.) Clairv. subsp. *solida*	.	.	.	.	.	.	.	.	.	.	.	.	.	.	.	D	.	.	.	.	.
Cotoneaster nebrodensis (Guss.) Koch	.	.	.	.	A	.	.	.	.	.	.	.	.	.	.	.	.	.	.	.	.
Crataegus azarolus L.*	.	.	.	.	CA	A	.	P	.	.	.	.	.	.	.	.	.	.	CA	CA	.
Crepis capillaris (L.) Wallr.	.	.	**P**	.	.	**P**	.	.	**P**	.	.	.	.	.	.	D	.	.	.	.	.
Crepis taraxacifolia Thuill. (= *Crepis vesicaria* L.)	.	.	.	.	**P**	**P**	.	**P**	.	.	.	.	.	.	.	**P**	.	.	D	.	.
Crocus longiflorus Raf.	.	.	.	.	.	.	A	.	.	.	.	.	.	.	.	.	.	.	**P**	.	.
Crocus neapolitanus (Ker Gawl.) Loisel. (= *Crocus imperati* Ten.)	.	.	.	.	.	.	.	.	RM	.	.	.	.	.	.	.	.	.	.	.	.
Crocus sativus L.*	A	A	.	.	.	.	.	.	D	.	.	CA	.	.	.	.	.	.	.	CA	.
Cucumis melo L. subsp. *melo* *	.	.	.	.	CA	.	.	.	.	.	.	.	.	.	A	.	.	.	CA	CA	.
Cucumis sativus L. subsp. *sativus**	.	.	.	.	CA	.	.	A	A	.	.	.	.	.	.	.	A	A	.	CA	.
Cucurbita maxima Duchesne subsp. *maxima**	A	CA	.	.	.	.	A	CA	CA	.	.	.	.	.	.	.	A	.	.	.	.
Cucurbita moschata Duchesne*	A	CA	.	.	.	.	CA	.	A	.	.	.	.	.	.	.	.	A	.	A	.
Cucurbita pepo L. subsp. *pepo**	.	.	.	.	CA	CA	.	CA	CA	.	.	.	CA	.	CA	.	.	A	.	CA	.
Cuminum cyminum L.*	.	.	.	.	A	.	.	.	.	.	.	.	.	.	.	.	.	.	.	.	.
Cydonia oblonga Mill.*	.	.	.	.	CA	A	A	.	CA	.	CA	**P**	**P**	**P**	CA	CA	CA	.	A	CA	.
Cynara cardunculus L. subsp. *flavescens* Wiklund	.	.	.	.	.	.	.	.	A	.	.	A	.	.	.	.	A	A	**P**	A	.
Cynara cardunculus L. subsp. *scolymus* (L.) Hegi*	.	.	.	A	A	.	.	A	CA	CA	CA	.	CA	CA	.	CA	CA	A	CA	CA	.
Daucus carota L. subsp. *sativus* (Hoffm.) Schübl. & G.Martens*	.	.	.	.	.	.	.	.	CA	.	.	.	.	.	.	.	.	.	.	.	.
Dianthus sylvestris Wulfen	.	.	.	.	.	.	.	.	.	.	.	RM	.	.	.	.	.	.	.	.	.
Diospyros kaki Thunb.*	.	.	.	.	A	.	.	.	A	.	.	.	CA	.	.	.	A	.	.	.	.
Dryopteris affinis (Lowe) Fraser-Jenk. subsp. *affinis*	.	.	.	.	.	.	.	.	.	.	.	.	.	.	.	A	.	.	.	.	.
Eruca vesicaria (L.) Cav.*	.	**P**	.	.	CA	**P**	**P**	**P**	**P**	**P**	**P**	**P**	**P**	**P**	**P**	**P**	**P**	**P**	**P**	**P**	.
Ervilia sativa Link (= *Vicia ervilia* (L.) Willd.)	.	.	.	.	NR	.	.	.	**P**	.	.	.	.	.	CA	**P**	.	.	.	.	.
Filipendula ulmaria (L.) Maxim.	.	.	.	.	**P**	.	.	.	.	.	.	.	.	.	.	NR	.	.	.	.	.
Fragaria viridis Weston subsp. *viridis*	.	.	.	.	.	.	.	.	.	.	.	.	.	.	.	**P**	**P**	D	.	.	.
Glyceria fluitans (L.) R. Br.	.	.	.	.	.	.	.	.	.	.	.	.	.	.	.	D	.	.	.	.	.
Glycine max (L.) Merr. subsp. *max*	.	.	.	.	CA	.	.	.	.	.	.	.	.	.	.	.	.	.	.	.	.
Glycyrrhiza echinata L.	.	.	.	.	.	.	.	.	.	.	.	.	.	.	.	.	.	A	.	.	.
Guizotia abyssinica (L.f.) Cass.	.	.	.	.	.	.	.	.	.	.	.	.	.	.	.	.	.	.	.	.	CA
Hordeum vulgare L. subsp. *vulgare**	.	CA	CA	.	CA	.	CA	CA	CA	.	CA	.	.	CA	.	.	A	.	A	CA	.
Hylotelephium maximum (L.) Holub subsp. *maximum* (=*Sedum maximum* (L.) Suter)	.	.	.	**P**	.	.	.	.	.	.	.	.	.	.	.	D	.	.	.	.	.
Hypochaeris cretensis (L.) Bory & Chaub.	A	A	.	.	.	.	.	.	.	.	.	.	.	.	.	**P**	.	**P**	**P**	.	.
Ipomoea batatas L.*	.	.	.	.	A	.	.	.	.	.	.	.	.	.	.	.	.	.	.	.	.
Jacobaea aquatica (Hill) G.Gaertn., B.Mey. & Scherb.										RM											
Jasminum officinale L.	.	.	.	.	.	.	.	.	.	.	.	.	.	.	.	.	.	.	CA	.	.
Juniperus phoenicea L.	.	.	.	.	.	.	.	.	.	.	.	.	.	.	.	.	.	.	.	RM	.
Knautia arvensis (L.) Coult.	**P**	**P**	.	.	**P**	.	**P**	.	**P**	RM	.	.	.	.	.	**P**	.	.	.	.	.
Lagenaria siceraria (Molina) Standl.*	.	.	.	.	.	.	.	.	.	.	.	.	.	A	.	.	.	A	.	.	.
Lathyrus odoratus L.	.	.	.	.	.	.	.	.	.	.	.	.	.	.	.	NR	.	.	**P**	.	.
Leontodon saxatilis Lam. (= *Leontodon crispus* Vill.)	.	.	.	.	.	.	.	.	.	.	.	.	.	.	.	NR	.	.	.	.	.
Leontodon siculus (Guss.) Nyman	.	.	.	.	.	.	.	.	.	A	.	.	.	.	.	.	.	.	**P**	.	.
Leontopodium nivale (Ten.) É.Huet & A.Huet ex Hand.-Mazz.	.	A	.	.	.	.	.	.	.	.	.	.	.	.	.	.	.	.	.	.	.
Lepidium latifolium L.	.	.	.	.	**P**	.	.	.	.	D	.	.	.	.	.	.	.	.	**P**	**P**	.
Lepidium sativum L. subsp. *sativum*	.	.	.	.	**P**	.	.	.	.	.	.	.	.	NR	.	.	.	.	.	CA	.
Levisticum officinale W.D.J.Koch	.	.	.	.	.	CA	.	.	.	.	.	.	.	.	.	.	.	.	.	.	.

Loncomelos narbonense (L.) Raf. (= *Ornithogalum narbonense* L.)	.	NR	.	.	.	.	.	.	.	.	.	.	.	.	.	**P**	.	.	.	.	.
Lupinus angustifolius L.*	.	.	.	.	A	.	.	.	.	.	.	.	.	.	.	.	**P**	.	.	**P**	.
Malus domestica (Suckow) Borkh.																			A		
Mantisalca salmantica (L.) Briq. & Cavill.	.	.	.	.	.	.	.	.	.	.	.	.	.	.	.	D	**P**	.	.	.	.
Mentha arvensis L.*	.	.	**P**	**P**	.	**P**	**P**	**P**	**P**	.	.	.	.	.	.	.	**P**	D	.	.	.
Mentha longifolia (L.) Hudson*	.	**P**	**P**	.	**P**	**P**	.	**P**	**P**	.	.	.	.	**P**	**P**	**P**	**P**	**P**	RM	.	.
Mentha microphylla K.Koch (=*Mentha spicata* subsp. *condensata* (Briq.) Greuter & Burdet)	.	.	.	.	.	.	.	.	.	.	.	.	.	.	.	.	.	NR	.	.	.
Mentha x piperita L.*	.	.	.	.	**P**	**P**	A	A	A	.	**P**	**P**	.	**P**	.	.	.	A	**P**	**P**	.
Montia fontana L.	.	.	.	.	.	.	.	.	.	.	.	.	.	.	.	A	.	.	.	.	.
Moricandia arvensis (L.) DC.	.	.	.	.	.	.	.	.	.	.	.	.	.	.	.	NR	.	.	**P**	**P**	.
Morus alba L.*	.	.	**P**	.	**P**	.	CA	**P**	CA	.	.	.	.	**P**	.	.	CA	CA	A	CA	.
Morus nigra L.*	.	.	.	.	RM	.	.	RM	CA	.	A	.	.	CA	.	.	CA	CA	A	CA	.
Myrtus communis L.	.	A	**P**	A	A	**P**	**P**	RM	**P**	**P**	**P**	**P**	**P**	**P**	**P**	**P**	.	**P**	**P**	**P**	.
Najas marina L.	.	.	.	.	.	.	.	.	.	.	.	.	.	.	.	.	.	.	.	.	RM/A
Nigella arvensis L.	.	.	.	.	A	.	.	.	.	.	.	.	.	.	.	**P**	.	.	.	.	.
Ocimum basilicum L.*	.	.	CA	.	CA	CA	CA	.	CA	.	A	.	CA	RM	.	.	CA	A	CA	CA	.
Oenothera biennis L.	.	.	**P**	.	.	.	.	.	.	.	.	.	.	.	.	.	D	.	.	.	.
Onopordum horridum Viv.	.	A	.	.	.	.	A	.	.	.	.	.	.	.	.	**P**	**P**	.	**P**	**P**	.
Ophrys sphegodes Mill.	.	.	.	.	.	.	.	.	.	.	.	.	.	.	.	.	.	.	.	A	.
Origanum majorana L.*	.	.	.	.	CA	.	.	.	CA	CA	CA	.	.	A	.	CA	CA	.	**P**	**P**	.
Origanum vulgare L. subsp. *viridulum* (Martrin-Donos) Nyman	.	A	.	.	.	.	A	.	A	.	.	.	.	**P**	**P**	**P**	**P**	**P**	**P**	.	.
Ornithogalum umbellatum L.	.	.	**P**	D	.	.	.	.	.	.	.	.	.	D	.	.	D	D	.	.	.
Oryza sativa L. subsp. *sativa*	A	**P**	.	.	**P**	.	A	.	CA	.	.	.	.	.	.	.	.	.	.	.	.
Oxalis acetosella L.	**P**	**P**	**P**	**P**	**P**	**P**	.	**P**	**P**	.	.	.	.	.	.	D	.	.	.	.	.
Panicum miliaceum L.	.	.	P	.	CA	.	.	.	CA	.	.	.	.	.	.	.	.	.	.	.	.
Papaver argemone L.																	A				
Papaver setigerum DC. (=*Papaver somniferum* subsp. *setigerum* (DC.) Arcang.)																	A				
Parietaria officinalis L.	**P**	**P**	**P**	.	.	.	**P**	**P**	**P**	**P**	**P**	**P**	**P**	**P**	**P**	**P**	**P**	RM	RM	.	.
Pastinaca sativa L. subsp. *sativa**	.	.	**P**	.	**P**	.	.	.	.	**P**	.	RM	**P**	**P**	A	A	.	.	.	.	.
Pelargonium graveolens L'Hér.	.	.	.	.	.	A	.	.	.	.	.	.	.	.	.	.	.	.	.	.	.
Persicaria hydropiper (L.) Delarbre	.	.	**P**	.	**P**	.	.	.	.	.	.	.	.	.	.	A	.	.	.	.	.
Petroselinum crispum (Mill.) Fuss*	.	.	CA	.	CA	CA	CA	.	CA	.	.	CA	.	CA	CA	CA	CA	CA	CA	P	.
Phaseolus coccineus L.	.	.	.	.	A	.	.	.	.	.	.	.	.	.	.	.	.	.	.	.	.
Phaseolus vulgaris L. subsp. *vulgaris**	.	.	.	.	CA	CA	.	.	D	.	.	.	.	.	.	.	A	A	A	CA	.
Phyteuma michelii All.	.	**P**	.	.	.	.	A	.	.	.	.	.	.	.	.	.	.	.	.	.	.
Pimpinella anisum L.*	.	.	CA	.	A	A	.	.	CA	.	CA	.	A	A	.	CA	.	A	.	CA	.
Pinus cembra L.	.	**P**	**P**	.	**P**	.	RM	.	.	.	.	.	.	.	.	.	.	.	.	.	.
Pistacia vera L.*	.	.	.	.	.	.	.	.	.	.	.	.	.	.	.	.	.	.	RM	A	.
Plantago media L.	**P**	**P**	**P**	.	.	**P**	.	.	**P**	.	.	.	.	.	**P**	**P**	**P**	.	.	A	.
Prunus armeniaca L.*	.	.	.	.	CA	.	CA	.	CA	.	.	.	CA	CA	.	.	CA	.	.	CA	.
Prunus cerasus L.*	.	.	CA	.	CA	**P**	CA	CA	CA	.	**P**	**P**	**P**	CA	.	.	CA	CA	.	.	.
Prunus domestica L.*	.	CA	.	.	CA	**P**	**P**	.	**P**	.	.	.	.	**P**	.	.	CA	CA	.	**P**	.
Prunus laurocerasus L.									CA												
Prunus persica (L.) Batsch.*	.	.	.	.	CA	**P**	CA	.	CA	.	.	.	CA	.	.	.	.	.	.	CA	.
Pseudopodospermum hispanicum (L.) Zaika, Sukhor. & N.Kilian	.	.	.	.	**P**	**P**	.	.	.	.	.	.	.	.	.	NR	.	.	**A**	.	.
Pseudopodospermum undulatum (Vahl) Zaika, Sukhor. & N.Kilian subsp. *deliciosum* (Guss.) Bartolucci, Galasso & F.Conti	.	.	.	.	.	.	.	.	.	.	.	.	.	.	.	.	.	NR	**P**	.	.
Pulmonaria officinalis L.	**P**	**P**	.	.	**P**	.	**P**	.	**P**	.	.	.	.	.	A	.	.	.	.	.	.
Punica granatum L.*	.	.	.	.	**P**	.	.	**P**	D	.	CA	CA	.	**P**	CA	.	CA	CA	A	**P**	.
Raphanus raphanistrum L. subsp. *sativus* (L.) Schmalh.*	.	.	.	.	CA	CA	.	.	CA	.	.	.	.	.	.	.	CA	.	.	CA	.
Rapistrum perenne (L.) All.*	.	.	.	.	.	.	.	.	.	.	.	.	.	.	.	.	CA	.	.	.	.
Rhaphiolepis bibas (Lour.) Galasso & Banfi	.	.	.	.	.	.	.	.	CA	.	.	.	.	.	.	.	.	.	.	.	.
Rheum rhabarbarum L.*	.	.	A	CA	CA	A	.	.	.	.	.	.	.	.	.	.	.	A	.	.	.
Ribes rubrum L.*	.	.	.	CA	CA	**P**	**P**	.	.	D	.	.	.	.	.	.	D	.	.	.	.
Ribes uva-crispa L.*	.	**P**	.	.	**P**	**P**	**P**	.	.	.	**P**	**P**	.	.	.	.	D	.	.	.	.
Rumex patientia L. subsp. *patientia*	.	.	.	.	.	.	.	.	.	.	.	.	.	.	.	.	.	.	.	A	.
Rumex scutatus L. subsp. *scutatus*	**P**	**P**	.	.	**P**	.	.	.	.	.	.	**P**	.	.	.	**P**	.	**P**	**P**	A	.
Ruta graveolens L.	.	.	**P**	.	**P**	**P**	CA	.	**P**	.	**P**	.	.	.	**P**	**P**	**P**	**P**	.	.	.
Salsola kali L.	.	.	.	.	.	.	.	.	.	.	.	.	.	.	.	RM	.	.	.	.	.
Salvia officinalis L.*	.	A	**P**	.	**P**	**P**	A	.	**P**	.	**P**	**P**	**P**	**P**	**P**	**P**	**P**	**P**	**P**	**P**	.
Salvia pratensis L.	.	**P**	**P**	**P**	.	**P**	**P**	**P**	**P**	**P**	.	.	.	**P**	**P**	RM	RM	.	.	.	.
Sambucus racemosa L. subsp. *racemosa*	**P**	**P**	.	**P**	**P**	.	.	.	.	.	.	.	.	.	.	.	A	.	.	.	.
Santolina chamaecyparissus L.	.	.	.	.	.	.	CA	.	.	.	.	.	.	.	.	.	.	.	.	.	.
Satureja hortensis L.*	.	.	CA	.	CA	.	.	**P**	.	.	CA	**P**	.	.	.	.	.	.	.	.	.
Schinus molle L.	.	.	.	.	.	.	.	.	.	.	.	.	.	CA	.	.	.	.	.	CA	.
subsp. *decumbens* (Guss.) Greuter (=*Podospermum laciniatum* subsp. *decumbens* (Guss.) Ge	.	.	.	.	.	.	.	.	.	.	.	D	.	.	.	**P**	.	.	**P**	.	.
Secale cereale L. subsp. *cereale**	.	.	CA	.	CA	CA	CA	CA	CA	.	.	.	.	.	.	.	.	.	.	.	.
Sicyos edulis Jacq. (=*Sechium edule* (Jacq.) Sw.)	.	.	.	.	CA	.	.	.	.	.	.	.	.	.	.	.	.	.	.	.	.
Silybum marianum (L.) Gaertn.	.	CA	**P**	.	**P**	.	**P**	.	**P**	.	**P**	**P**	**P**	**P**	**P**	**P**	**P**	**P**	**P**	**P**	.
Solanum lycopersicum L.*	.	.	.	.	.	.	.	CA	CA	.	CA	CA	.	.	CA	.	CA	.	.	.	.
Solanum melongena L. subsp. *melongena**	.	.	.	.	.	.	.	.	CA	.	.	.	.	.	.	.	.	.	.	.	.
Solanum tuberosum L.*	.	CA	CA	.	CA	CA	A	CA	CA	.	CA	.	.	CA	CA	.	A	.	A	CA	.
Sonchus arvensis L.	.	**P**	.	.	**P**	.	.	**P**	**P**	.	**P**	**P**	.	.	**P**	.	**P**	**P**	A	**P**	.
Sorghum bicolor (L.) Moench subsp. *bicolor**	.	.	.	.	CA	.	.	CA	CA	.	.	.	.	.	.	.	.	.	.	.	.
Spinacia oleracea L.*	.	.	.	.	CA	A	.	CA	CA	.	.	.	.	.	.	.	.	.	.	CA	.
Tanacetum balsamita L.	CA	CA	CA	.	CA	A	CA	CA	CA	.	CA	CA	CA	CA	CA	.	.	.	CA	CA	.
Tanacetum parthenium (L.) Sch.-Bip.	.	.	**P**	.	.	.	CA	.	**P**	.	**P**	**P**	**P**	**P**	.	**P**	.	.	.	**P**	.
Taraxacum fulvum Raunk.	.	.	.	.	.	.	.	.	.	.	.	.	.	A	.	A	.	A	A	.	.
Taraxacum minimum (brig. ex Guss.) N.Terracc.	.	.	.	.	.	.	.	.	.	.	.	.	.	.	.	**P**	.	.	D	**P**	.
Thalictrum minus L.	.	.	.	.	.	.	.	.	.	.	.	.	.	.	.	NR	.	.	.	.	.
Thymus pulegioides L.	**P**	**P**	**P**	.	.	**P**	**P**	.	**P**	.	.	.	.	.	.	RM	RM	RM	.	.	.
Thymus vulgaris L. subsp. *vulgaris*	.	**P**	**P**	.	.	.	**P**	.	**P**	**P**	.	.	.	.	.	CA	.	.	.	A	.
Tragopogon pratensis L.	**P**	**P**	**P**	**P**	**P**	**P**	**P**	**P**	**P**	**P**	**P**	**P**	**P**	**P**	**P**	NR	.	.	.	.	.

Triticum aestivum L.*	.	.	CA	.	CA	CA	CA	.	CA	.	CA	.	.	.	.	A	.	CA	.	CA	.
Triticum aestivum L. subsp. *spelta* (L.) Thell.*	.	.	.	.	A	.	.	.	.	.	.	.	.	.	.	.	A	.	.	.	.
Triticum turgidum L. subsp. *dicoccon* (Schrank) Thell.*	.	.	.	.	.	.	.	.	CA	.	.	.	.	.	.	.	.	.	.	.	.
Triticum turgidum L. subsp. *durum* (Desf.) Husn.*	.	.	.	.	.	.	.	.	.	.	.	.	.	.	A	.	CA	CA	.	CA	.
Tropaeolum majus L.	.	.	.	.	.	.	.	.	.	.	.	.	.	.	.	CA	.	.	**P**	.	.
Ulex europaeus L. subsp. *europaeus*	.	.	.	.	.	.	.	.	.	.	.	.	.	.	.	D	.	.	.	.	.
Valeriana officinalis L.	.	.	.	.	.	.	**P**	.	**P**	.	**P**	.	.	**P**	.	D	**P**	**P**	.	.	.
Vanilla planifolia Jacks. ex Andrews*	.	.	.	.	.	.	.	A	.	.	.	.	.	.	.	.	.	.	.	A	.
Vicia faba L.*	.	CA	.	.	CA	.	CA	CA	CA	.	A	.	CA	CA	CA	.	CA	CA	CA	CA	.
Vicia lens (L.) Coss. & Germ. subsp. *lens** (= *Lens culinaris* Medik.)	.	CA	.	.	A	.	.	.	CA	.	.	CA	CA	.	.	.	A	A	A	CA	.
Vicia villosa Roth	.	.	.	.	.	.	.	.	.	.	.	.	.	.	.	**P**	A	.	.	.	.
Vigna unguiculata (L.) Walp.*	.	.	.	.	.	.	.	.	.	.	.	.	.	.	.	.	.	CA	.	A	.
Viola aethnensis (DC.) Strobl subsp. *aethnensis*	.	.	.	.	.	.	.	.	.	.	.	.	.	.	.	.	A	.	.	.	.
Vitis vinifera L.*	CA	**P**	.	.	CA	**P**	**P**	**P**	**P**	.	**P**	.	**P**	**P**	.	**P**	**P**	**P**	**P**	**P**	.
Wisteria sinensis (Sims) Sweet*	.	.	.	.	.	.	.	.	CA	.	.	.	.	.	.	.	.	.	A	.	.
Zea mays L.*	A	A	CA	.	CA	CA	A	CA	CA	.	CA	.	.	.	CA	.	CA	CA	.	CA	.
Ziziphus jujuba Mill.*	.	.	.	.	**P**	.	.	.	**P**	.	.	.	.	CA	A	**P**	CA	**P**	CA	**P**	.
