# Supplementary material for: Design a Database of Italian Vascular Alimurgic Flora (AlimurgITA): Preliminary Results
Source: Plants (Basel). 2021 Apr 10;10(4):743. doi: 10.3390/plants10040743 (PMC8069721; doi:10.3390/plants10040743)
Supplement: Supplementary file 1 [file plants-10-00743-s001.zip › Paura et al_Database alimurgic flora Italy 2021_Supplementary Materials_Table S2.pdf]

Supplementary Materials Table S2. List of the 358 selected texts (*data not yet entered in the database).

N.	Text	Area
1	AA.VV., *Biodiversità coltivata nel Parco Nazionale Dolomiti Bellunesi* ; Parco Nazionale Dolomiti Bellunesi, Feltre (BL), Italy, 2006.	Veneto
2	Accogli, R.; Gambetta G.; Medagli P. *Erbe spontanee della Basilicata. Guida al riconoscimento e all'utilizzo delle piante alimentari tradizionali* ; Ed. Grifo (Cavallino), 2017; pp. 192.	Basilicata
3	Accogli, R.; Marchiori, S. *Ricerche etnobotaniche nell'agro di Fasano* (Brindisi-Puglia). IV Convegno Nazionale "Piante Mediterranee. Le potenzialità del territorio e dell'Ambiente" Marina di Nova Siri (MT). 7–10 ottobre 2009**;** pp. 166.	Apulia
4	Accogli, R.; Marrese M.; Medagli P. *Erbe spontanee della capitanata. Guida al riconoscimento e all'utilizzo delle piante alimentari tradizionali* . Ed. Grifo (Cavallino)**,** 2017; pp. 192.	Apulia
5	Accogli, R.; Medagli P. *Erbe spontanee della Calabria. Guida al riconoscimento e all'utilizzo delle piante alimentari tradizionali;* Ed. Grifo (Cavallino), 2018; pp. 192.	Calabria
6	Accogli, R.; Medagli P. *Erbe spontanee salentine. Guida al riconoscimento e all'uso delle piante alimentari tradizionali;* Ed. Grifo (Cavallino)**,** 2014; pp. 208.	Apulia
7	Accogli, R.; Medagli, P.; Perrino, E.V. *Erbe spontanee in Terra di Bari. Guida al riconoscimento e all'utilizzo delle piante alimentari tradizionali* ; Ed. Grifo (Cavallino), 2015; pp. 192.	Apulia
8	Aleo, M.; Azzaro, D.; Cambria, S.; Bazan, G. Indagini etnobotaniche nel territorio di Paceco (Sicilia Occidentale). *Naturalista sicil.* **2020,** S. IV, *XLIV* (1-2), 169-208.	Sicily
9	Aleo, M.; Cambria, S.; Bazan, G. Tradizioni etnofarmacobotaniche in alcune comunità rurali dei Monti di Trapani (Sicilia occidentale) *Quad. Bot. Amb. Appl.* **2013,** *24* , 27-48.	Sicily
10	Aliotta, G. Edible Wild Plants in Italy. *Inform. Bot. Ital.* **1987,** *19* (1), 17–30.	Italy
11 *	Aliotta, G.; Salerno, C. *Le piante alimentari in Campania dalle origini al Duemila* EDIZIONI ATHENA NAPOLI, 1995.	Campania
12	Altobelli, A.; Braut, R.; Boriani, M. L'erbariol – Piante spontanee di usi alimentare nella Bisiacaria; Associazione Culturale Bisiaca, 2013; pp. 1-47.	Friuli Venezia Giulia
13	Ansaldi, M.; Tomei, P.E. Specie vegetali selvatiche e coltivate di uso medicinale nella tradizione popolare delle Apuane settentrionali. *Atti Soc. Tosc. di Sci. Nat. Mem. Serie B,* **1997**, *104,* 19-34.	Tuscany
14	Appi, E.; Appi, R.; Pagnucco, A.; Pagnucco, D. *Le piante nell'uso popolare in Friuli . Terapia e Cucina;* Ed. Concordia Sette: Pordenone, Italy, 1979; pp. 120.	Friuli Venezia Giulia
15	Arcidiacono S.; Pavone, P.; Salmeri, C. Specie spontanee usate per l'alimentazione umana nel territorio etneo (Sicilia Orientale). *Giornale Botanico Italiano* **1995,** 129(2), 149.	Sicily
16	Arcidiacono, S. *Etnobotanica Etnea - Le piante selvatiche e l'uomo;* Ed. Danaus, 2016; pp. 150.	Sicily
17	Arcidiacono, S. Flora popolare nel territorio di Bronte (CT). In *Etnobotanica nella Provincia di Catania con Atti del Convegno "Andar per verdure";* Nuova Zangara Stampa Editrice: Linguaglossa, Italy, 2002.	Sicily
18	Arcidiacono, S. *Le verdure spontanee dell'Etna Catania* . Azienda Provinciale del Turismo-Pro Loco Linguaglossa 1998.	Sicily
19	Arcidiacono, S.; Costa, R.; Marletta, G.; Pavone, P.; Napoli, M. Usi popolari delle piante selvatiche nel territorio di Villarosa (EN – Sicilia Centrale) *Quad. Bot. Amb. Appl* . **2010,** *21* , 95-118.	Sicily
20	Arcidiacono, S.; Napoli M.; Oddo G.; Pavone P. Piante selvatiche d'uso popolare nei territori di Alcara li Fusi e Militello Rosmarino (Messina N-E Sicilia). *Quad. Bot. Amb. Appl.* **2007,** *18* , 105–146.	Sicily
21	Arcidiacono, S.; Napoli, M.; Pavone, P. Piante spontanee d'uso popolare nel territorio di Bronte (Catania). *Quad. Bot. Amb. Appl.* **2003**, *14* , 151-172.	Sicily
22	Arcidiacono, S.; Pavone, P. Erbe spontanee commestibili del territorio etneo. *Boll. Acc. Gioenia Sci. Nat.* **1994**, *27* (346), 461-588.	Sicily
23	Arcidiacono, S.; Pavone, P.; Salmeri, C. Erbe commestibili dell'Etna e loro valenza economica. Accademia della cucina, Conv. Intern. di Studi "Funghi, Tartufi e erbe mangerecce" L'Aquila 1995.	Sicily
24	Arietti, N. *La flora economica e popolare del territorio Bresciano;* Fratelli Geroldi ed., Brescia, Italy, 1974; pp. 186.	Lombardia

25	Arietti, N. *La nostra flora nell'economia domestica. Note pratiche sulla utilizzazione dei vegetali spontanei nella Provincia di Brescia con particolare trattazione degli usi locali e delle denominazioni volgari bresciane e italiane;* La Scuola: Brescia, Italy, 1941.	Lombardia
26	Arrigoni, P.; Cerni, S.; Simeone, P.; Sarti, S. *Le buone erbe della campagna riminese;* Editore: Provincia di Rimini - Assessorato Attività Produttive Servizio Agricoltura e Alimentazione II Edizione, 2002; pp. 205.	Emilia Romagna
27	Arrigoni, P.C.; Cerni S.; Piccari Ricci, S.; Sarti S. *Le buone erbe della campagna Riminese;* Provincia di Rimini, Rimini, Italy, 1996; pp. 200.	Emilia Romagna
28	Atzei, A.D. *Le piante nella tradizione popolare della Sardegna* ; C. Delfino Editore: Sassari, Italy, 2003; pp. 596.	Sardinia
29	Atzei, A.D.; Orioni, S.; Sotgiu, R. Contributo alla conoscenza degli usi etnobotanici nella Gallura (Sardegna). *Boll. Soc. Sarda Sci. Nat.* **1991**, *28* , 137-177.	Sardinia
30	Bagnoli, G.; di Soccio, C. *Molise in Verde; Non c'è erba volta in su che non abbia sua virtù;* Esse Media editrice, 2005; pp. 66.	Molise
31	Bagnoni, V.; Igneri, E.; Turini M. *Erbe di casa mia. Ricette del monte Pisano;* Felici Ed. 2008; pp. 206.	Tuscany
32	Ballero, M.; Bruni, A.; Sacchetti, G.; Mossa, L.; Poli F. Indagine etnofarmacobotanica del territorio di Arzana (Sardegna orientale); *Ann. Bot.* **1994,** *52* , 489-500.	Sardinia
33	Barbagallo, C.; Furnari F. *Flora officinale del territorio di Caltagirone (Catania);* Ed. Succ. Fusi, Pavia, 1967.	Sicily
34	Belcari, M.S. Una ricerca etnobotanica nel territorio di S. Maria a monte (Toscana): primo contributo Rivista di preistoria, etnografia, storia naturale. *Ist. Stor. Lucch* . **2012**, *VII-X* **,** 7-47.	Tuscany
35	Bellia, G.; Pieroni, A. Isolated, but transnational: the glocal nature of Waldensian ethnobotany, Western Alps, NW Italy. Journal of ethnobiology and ethnomedicine **2015,** *11* (1), 37.	Piemonte
36	Bellomaria, B. Le piante di uso popolare nel territorio di Camerino (Marche) *Arch. Bot. Biogeogr. Ital.* **1982,** *58* (3-4), 1–27.	Marche
37	Bellomaria, B.; Della Mora, L. Novità nell'uso delle piante officinali per la zona di Matelica (Macerata) anche in confronto con altre zone delle Marche Arch. Bot.Biogeogr.Ital., **1985,** *61* (1-2), 51-81.	Marche
38	Bellomaria, B.; Lattanzi, E. Le piante del territorio di Cupra Marittima (Marche) attualmente usate nella medicina popolare *Arch. Bot. e Biogeograf. Ital.* **1982,** *58* (3-4): 1-19.	Marche
39	Benvenuti, R.; Covarelli, A.; Storai, R. *Le erbe officinali del territorio: curiosità e ricette di cucina della Comunità Montana Val di Bisenzio* ; Ed. Comunità Montana Val di Bisenzio, 1996.	Tuscany
40	Bernardo, L. *Fiori e piante del Parco del Pollino;* Ed. Prometeo, Castrovillari, 2000.	Basilicata
41	Bianchi, A.; Gallifuoco, G. Farmacopea Popolare del Gargano. Natural **2004**, *32* , 54-66.	Apulia
42	Bianco V.; Mariani R.; Santamaria P. Piante spontanee nella cucina tradizionale molese. Storie, curiosità e ricette; Bari, Ed. Levante 2009; pp. 276.	Apulia
43	Bianco, V.V. Specie erbacee della flora infestante pugliese utilizzabili come ortaggi e piante da condimento. *Atti e relazioni dell'Accademia pugliese delle Scienze* . Fasano, Grafischena. 1989; 46 tomo II, 11-27.	Apulia
44	Bianco, V.V. Specie erbacee spontanee eduli della flora pugliese. In Macchia Francesco (a cura di) *La flora e la vegetazione spontanea della Puglia nella scienza, nell'arte e nella storia: atti del Convegno* , Bari, 22-23 maggio 1993. Fasano: Schena, 1997; pp. 61-83.	Apulia
45	Bianco, V.V.; Macchackova M. Specie spontanee della flora italiana utilizzate come ortaggi e piante da condimento. in: Atti VI Giornate Scientifiche SOI, Spoleto, 23-25 Aprile 2002. Spoleto; pp. 435-436.	Italy
46	Biscotti, N. *Botanica delle erbe eduli . Peregrinazioni fitoalimurgiche . Dal Gargano alle Puglie;* Ed. Centro Grafico Francescano, Foggia 2013; pp. 504.	Apulia
47	Biscotti, N.; Bonsanto, D. *Vie erbose. Le erbe selvatiche nelle bioculture alimentari mediterranee;* Editore: Centro Grafico 2020; pp. 560.	Apulia
48	Biscotti, N.; Bonsanto, D.; Del Viscio, G. The traditional food use of wild vegetables in Apulia (Italy) in the light of Italian ethnobotanical literature. *Italian Botanist* **2018,** *5* , 1–24.	Apulia
49	Biscotti, N.; Del Viscio, G.; Bonsanto D. Indagine etnobotanica sull'uso alimentare tradizionale di piante selvatiche in un comprensorio montano della Regione Puglia (Subappennino Dauno, Provincia Di Foggia) Atti Soc. Tosc. Sci. Nat. Mem., Serie B, 2018, 125, 17-29.	Apulia

50	Biscotti, N.; Pieroni, A. The hidden Mediterranean diet: Wild vegetables traditionally gathered and consumed in the Gargano area, Apulia, SE Italy. *Acta Soc. Bot. Poloniae* **2015,** *84* (3), 327–338.	Apulia
51	Bisio, A.; Minuto, L. Il Preboggiun: leggenda e tradizione di una miscela di erbe selvatiche. In Il Cibo e il Corpo: dal cibo alla cultura, dalla cultura al cibo; Ed. Erga: Genova, 1999; pp. 197-229.	Liguria
52	Bisio, A.; Minuto, L. *The Prebuggiun.* In: Pieroni, A. (a cura di), *Erbi Boni, Erbi degli Streghi* , Experiences Verlag, Colonia, 1998; pp. 34-46.	Liguria
53	Boari, F.; Cefola, M.; Di Gioia, F.; Pace, B.; Serio, F.; Cantore, V. Effect of cooking methods on antioxidant activity and nitrate content of selected wild Mediterranean plants Int J Food Sci Nutr 2013, 1–7.	Mediterranean
54	Bonomo, R.; Trapani S. Piante officinali nelle Egadi (Favignana e Levanzo) L*av. Ist. Bot. Giard. Col.* Palermo **1973,** *25* , 195-238.	Sicily
55	Borgonovo, G.; Caimi, S.; Morini, G.; Scaglioni L.; Bassoli A. Taste active compounds in a traditional Italian food: 'lampascioni' *Chem. Biodivers.* **2008,** *5* , 1184–1194.	Italy
56	Bruni, A.; Ballero, M.; Poli F. Quantitative ethnopharmacological study of the Campidano Valley and Urzulei district, Sardinia, Italy *Journal of Ethnopharmacology* **1997,** *57* , 97-124.	Apulia
57	Bucci, A. *Gli erbi a Camaiore;* 1999; pp. 27-32.	Tuscany
58	Calzolari, E. Le erbe spontanee nelle tradizioni gastronomiche del promontorio del Caprione (La Spezia). In *Erbi boni, erbi degli streghi;* Pieroni, A., Ed.; Experiences Verlag, Cologne, Germany, 1998; pp. 24-33.	Liguria
59	Camangi, F. *Erbe Selvatiche Alimentari delle Apuane e della Lunigiana;* Ceccotti Ed., Massa, 1999.	Tuscany
60	Camangi, F.; Bettini, D.; Cecchelli, G.; Santoro, A.; Stefani, A. Piante spontanee d'uso alimentare: viaggio alla scoperta della cucina povera a partire dalla tradizione popolare volterrana (Toscana); Atti 105° Congresso Nazionale S.B.I. Milano. 25-28/8/2010 2010; p. 108.	Tuscany
61	Camangi, F.; Stefani A. *L'orto dei semplici nell'Eremo di Santa Caterina a Rio nell'Elba Frutta antica e piante spontanee nella tradizione alimentare elbana;* Ed. ETS, 2014; pp. 270.	Tuscany
62	Camangi, F.; Stefani A.; Giambastiani M. Appunti fitoalimurgici: la "zuppa matta" di Piegaio (Val Pedogna - Lucca - Toscana). A*tti dell'Istituto per la documentazione sul castagno e la ricerca forestale* **2005,** 85-98.	Tuscany
63	Camangi, F.; Stefani, A. Il castagno nella tradizione popolare. Provincia di Lucca e aree contermini. In: Le cultivar di castagno della provincia di Lucca - I parte. *Atti dell'Istituto per la documentazione sul castagno e la Ricerca Forestale (IRF)* **2011**, *2* , 61-64.	Tuscany
64	Camangi, F.; Stefani, A. Tradizioni Phytoalimurgiche in Toscana: Le piante selvatiche nella preparazione delle zuppe. *Riv. Preist., Etnogr. St. natur. Istituto Storico Lucchese* **2004,** 2 (1).	Tuscany
65	Camangi, F.; Stefani, A.; Lippi, A. 100 Piante spontanee d'uso alimentare del territorio di Capannori; Ed. Biolabs, 2009; pp. 320.	Tuscany
66	Camangi, F.; Stefani, A.; Lippi, A.; Tomei, P.E. *Piante selvatiche di uso alimentare nella tradizione popolare della Garfagnana; erbe buone ed erbe cattive* . Ed. Studio d'Arte fotografica Comunita Montana della Garfagnana, 2007; pp. 94.	Tuscany
67	Camangi, F.; Stefani, A.; Sebastiani, L. *Etnobotanica in val di Vara. L'uso delle piante nella tradizione popolare;* Ed. S.l., Biolabs, 2009.	Liguria
68	Camangi, F.; Stefani, A.; Sebastiano L. *Piante e folclore nelle Valli di Zeri in Lunigiana;* Ed. ETS, 2013; pp. 224.	Tuscany
69	Camangi, F.; Stefani, A.; Tomei P.E. *Tradizioni etnobotaniche nelle foreste Casentinesi;* Parco Nazionale Foreste Casentinesi, 2003.	Tuscany
70	Camangi, F.; Stefani, A.; Tomei, P.E. Tradizioni etnofarmacobotaniche nei comuni di Poppi e Bibbiena (Arezzo - Toscana). *Atti Soc. Tosc. Sci. Nat., Mem* . **2003,** Serie B, 1-110.	Tuscany
71	Camangi, F.; Tomei, P.E. *Piante medicinali nella tradizione popolare del Capannorese* Ed. La Grafica Pisana, 1999; pp. 171.	Tuscany
72	Camangi, F.; Tomei, P.E. Tradizioni etno-farmacobotaniche nella provincia di Livorno: il territorio della Valle Benedetta Inf. Bot. Ital. 2003, 35 (1), 41-54.	Tuscany
73	Camangi, F.; Uncini Manganelli R.E. L'etnobotanica nel territorio di Capannori: Stato delle conoscenze e nuove acquisizioni. In Tomei P.E., Kugler P.C. (curatori) *Aspetti biologici del Territorio del Comune di Capannori,* "Studi Capannoresi", III, Comune di Capannori, 1999; pp. 177-224.	Tuscany

74		Camangi, F.; Uncini Manganelli, R.E. Indagine etno-botanica nel pistoiese: il territorio di Collodi (PT). *Boll. Orto Bot. Lucca* **2004,** *3* (1), 19-39.	Tuscany
75		Camangi, F.; Uncini Manganelli, R.E. La cornucopia della natura: piante alimentari nella tradizione popolare del Monte Pisano. In *Monte Castellare – Valle delle Fonti: due aree protette dei Monti Pisani, aspetti naturalistici e storici.* Felici Editore: Pisa, Italy, 2000; pp. 45-52.	Tuscany
76		Camangi, F.; Uncini Manganelli, R.E. *La scoperta delle piante medicinali nella tradizione popolare* ; Felici Ed. 2002; pp. 35-103.	Tuscany
77		Camarda, I. Ricerche etnobotaniche nel Comune di Dorgali (Sardegna centro- occidentale). *Boll. Soc. Sarda Sci. Nat.* **1990,** *27* , 147-204.	Sardinia
78		Camarda, I.; Carta, L.; Vacca, G.; Brunu, A. Les plantes alimentaires de la Sardaigne: un patrimoine ethnobotanique et culturel d'ancienne origine. *Fl. Medit.* **2017**, *27*, 77-90.	Sardinia
79		Camarda, I.; Piras, G. Ethnobotanical knowledge in the agropastoral World of Putifigari village (Sardinia, Italy). *Delpinoa* **2003,** *45* , 213-220.	Sardinia
80		Caneva, G.; Pontrandolfi, M.A.; Fascetti, S. *Le piante alimentari spontanee della Basilicata;* Cons. Reg. Basilicata, Ufficio Stampa, 1997.	Basilicata
81		Cappelletti, E.M.; Cirio, M. E.; Mutti, L. L'uso delle piante officinali nella medicina popolare del Feltrino (Belluno). *Atti Ist. Veneto Sci., Lett. Arti* **1979**, *137* , 113-131.	Veneto
82		Cappelletti, E.M.; Trevisan, R.; Folletto, A.; Cattolica, P.M. Le piante utilizzate in medicina popolare in due vallate trentine: Val di Ledro e Val dei Mocheni *Studi Trentini Sci. Nat.* **1981,** *58* , 119-140.	Trentino Alto-Adige
83		Casoria, P.; Menale, B.; Muoio, R. Muscari comosum, Liliaceae, in the food habits of South Italy. *Economic Botany* **1999,** *53* , 113-117.	Southern Italy
84		Catanzaro, F. Piante officinali dell' isola di Pantelleria. *Fitoterapia* **1970,** *2* , 66-83.	Sicily
85		Catanzaro, F. Piante officinali dell' isola di Pantelleria. *Webbia* **1968,** *23* , 135-148.	Sicily
86		Certa, G. L'uso tradizionale delle piante nella comunità rurale di Chiusa Sclafani (Palermo). *Quad. Bot. Ambientale Appl.* **2020,** *31* , 3-14.	Sicily
87	*	Ceruti, A. *Piante medicinali e alimentari* ; Ed. Loescher, Torino, 1957.	Italy
88		Chiarungi, S.; Minuto, G. *Sette Buone Erbe Cellesche. Il Prebuggiun della Tradizione Comune di Celle Ligure* ; 2012; pp. 63.	Liguria
89		Chiavoni, M.; Raffo, E. S.; Taglialatela, A. *Ricerca etnobotanica nella Provincia di Grosseto.* Tip. La Stampa: Grosseto, Italy, 1994.	Tuscany
90		Chimenti Signorini, R.; Fumagalli, M. Indagine etnofarmacobotanica nella Valtournanche (Val d'Aosta). *Webbia* **1983,** *37*, 69-94.	Aosta Valley
91		Chiovenda-Bensi, C. Florula Medicinale delle Cinque Terre. *Webbia,* **1960,** *15* , 631-641.	Liguria
92		Chiovenda-Bensi, C. Piante medicinali nell'uso tradizionale della valle d'Ossola. *Atti Acc. Ligustica Sci. Lett. Genova* **1955,** 32-52.	Piemonte
93		Chiovenda-Bensi, C. Tradizioni e Usi fitoterapici popolari, la Valsesia. *Atti Acc. Ligustica Sci. Lett. Genova* **1957,** *13* , 190-205.	Piemonte
94		Clemente, P.; Giusti, M.E.; Di Pasquale, C. *Verso il Museo della Piana (Capannori – LU);* Progetto RUBIA ICA 3-2002-10055, 2005.	Tuscany
95		Coassini Lokar, L.; Poldini, L. Herbal remedies in the traditional medicine of the Venezia Giulia region (North East Italy). *Journal of Ethnopharmacology* **1988,** *22* , 231-278.	Friuli Venezia Giulia
96		Coassini Lokar, L.; Poldini, L.; Angeloni Rossi, G. Appunti di etnobotanica del Friuli-Venezia Giulia. *Gortania* **1982**, *4* , 101-151.	Friuli Venezia Giulia
97		Comin, S.; Pucciarelli, B. *Il mondo di Bepi Salon. Ricordi, ricette, erbe e funghi nella storia di un grande ristoratore della Carnia;* Camera di Commercio, Industria, Artigianato e Agricoltura di Udine, 2005; pp. 189.	Friuli Venezia Giulia
98		Corbetta, F. *99 modeste, umili, saporosissime piante spontanee mangerecce;* Edagricole: Bologna, Italy, 1991; pp. 181.	Italy
99		Cornara, L. *Il finocchio di mare (Crithmum maritimum L.) Caratteristiche e usi di una alofita della riviera ligure.* I Quaderni della Massocca, Litografia Conti: La Spezia, Italy, 2007.	Liguria
100		Cornara, L.; La Rocca, A.; Girani, A.; Gardella, P. L. *Erbe e tradizioni del Golfo Paradiso [Herbs and traditions of the Golfo Paradiso]* .Ed. Araba Fenice, 2013; pp. 208.	Liguria
101		Cornara, L.; La Rocca, A.; Marsili, S.; Mariotti, M.G. Traditional uses of plants in the Eastern Riviera (Liguria, Italy). *Journal of Ethnopharmacology* **2009,** *125* , 16–30.	Liguria

102		Cornara, L.; La Rocca, A.; Terrizzano, L.; Dente, F.; Mariotti, M. G. Ethnobotanical and phytomedical knowledge in the North-Western Ligurian Alps. *Journal of Ethnopharmacology* **2014**, *155* , 463-484.	Liguria
103		Cornara, L.; La Rocca, A.; Mariotti, M. *Andare per erbe. Piante e tradizioni della riviera spezzina;* Eds Ligurpress, Genova, Italy, 2011; pp. 107.	Liguria
104		Corsi, G.; Gaspari, G.; Pagni, A.M. L'uso delle piante nell'economia domestica della Versilia collinare e montana. *Atti Soc. Tosc. Sci. Nat., Mem.* **1980,** Serie B, *87* (1), 309-386.	Tuscany
105		Corsi, G.; Pagni, A.M. *Piante selvatiche di uso alimentare in Toscana;* Pacini Eds.: Pisa, Italy, 1979.	Tuscany
106		Corsi, G.; Pagni, A.M. Studi sulla flora e vegetazione del Monte Pisano (Toscana Nord-Occidentale). V. Le piante spontanee nella alimentazione popolare *Atti Soc. Tosc. Sci. Nat., Mem* . **1979,** Serie B, *86* (5) 79-101.	Tuscany
107	*	Costantini, G. *Litùn e Lidùn, erbucce primaverili mangerecce nella tradizione popolare friulana.* Rivista "Lares", Roma, 1941.	Friuli Venezia Giulia
108		Cucinotta, F.; Pieroni, A. 'If you want to get married, you have to collect virdura': the vanishing custom of gathering and cooking wild food plants on Vulcano, Aeolian Islands, Sicily *Food Cult.* **2018** Soc. 21, 539–567. doi: 10.1080/15528014.2018.1481263	Sicily
109		D'Africa, G. Relazione su una indagine erboristica condotta nell'Abruzzo-Molise. *EPPOS* **1963,** *8* , 379-387.	Abruzzi
110		De Bellis, A. L'uso popolare delle erbe spontanee in Val d'Orcia. *Erboristeria Domani* **200**8, *3,* 60-65.	Tuscany
111		De Feo, V.; Aquiro, R.; Menghini, A.; Ramundo, E.; Senatore, F. Traditional phytotherapy in the Peninsula Sorrentina, Campania, Southern Italy. *Journal of Ethnopharmacology* **1992,** *36,* 113-125.	Campania
112		De Feo, V.; Senatore F. Medicinal plants and phytotherapy in the Amalfitan Coast, Salerno Province, Campania, Southern Italy. *Journal of Ethnopharmacology* **1993,** *39,* 39-51.	Campania
113	*	De Martis Murranca, F. *Le piante nella tradizione popolare sarda;* EDISAR: Cagliari, Italy, 1992.	Sardinia
114		De Rosa, A.; Napolitano, B.; don Picariello, G. *Piante alimurgiche della Bassa Irpinia. Riconoscimento, diffusione ed utilizzazione gastronomica delle erbe selvatiche commestibili del Baianese e del Lauretano* . Youcanprint Self-Publishing, 2015; pp. 292.	Campania
115		De Simoni, E.; Guarrera, P.M. Indagine etnobotaniche nella provincia di Teramo. *Quad. Bot. Amb. e Appl.* **1994**, *5,* 3-10.	Abruzzi
116		Della Monaca, G.; Roselli, D.; Tosi, G. La Botanica popolare del Monte Argentario. In *Geobotanica e Etnobotanica del Monte Argentario;* Laurum Editore, 2001; pp. 123-245.	Tuscany
117	*	Dell'Aquila, G.; Sicilia, S. *Flora spontanea a Cisternino.* Ed. Schena 2010; pp. 142.	Apulia
118		Di Massimo, S; Di Massimo, M. *Ritorno alle Radici. Le piante spontanee per l'alimentazione e la salute.* Ed. Aboca, 2015; pp. 415.	Italy
119		Di Novella, R.; Di Novella, N.; De Martino, L.; Mancini, E.; De Feo, V. Traditional plant use in the National Park of Cilento and Vallo di Diano, Campania, Southern Italy. *Journal of Ethnopharmacology* **2013**, *145,* 328–342.	Campania
120		Di Renzo, E. Alimentazione, salute, età in un contesto specifico di studio. Le pratiche fitoalimurgiche nel comprensorio di Oratino (CB). In Longo, E, Cedri, C, Giustini, M. (Eds.) *Invecchiare oggi: una sfida per il domani. Risultati del Progetto Europeo CHANGE (Care of Health Advertising New Goals for Elderly people)* . Istituto Superiore di Sanità. Roma, 3 dicembre 2010. Atti. Roma: Istituto Superiore di Sanità 2011; pp. 25-38.	Molise
121		Di Tizio, A.; Łuczaj, Ł. J.; Quave, C. L.; Redžić, S.; Pieroni, A. Traditional food and herbal uses of wild plants in the ancient South-Slavic diaspora of Mundimitar/Montemitro (Southern Italy). *J. Ethnobiol. Ethnomed.* **2012**, *8* :21.	Molise
122		Disciglio, G.; Tarantini, A.; Frabboni, L.; Gagliardi, A.; Giuliani, M.M.; Tarantino, E.; Gatta, G. Qualitative characterization of cultivated and wild edible plants: mineral elements, phenols content and antioxidant capacity. *Italian Journal of Agronomy* **2017**, *12,* 10-36.	Italy
123		Ditonno, N.; Lamusta, S. Sapori e aromi da piante e frutti spontanei della Puglia peninsulare. Ed. Amici della "A. De Leo": Brindisi, Italy, 1997; pp. 576.	Apulia
124		Dreon A.L.; Paoletti M.G. The wild food (plants and insects) in Western Friuli local knowledge (Friuli-Venezia Giulia, North Eastern Italy). *Contributions to Natural History* **2009,** *12* (1), 461-488.	Friuli Venezia Giulia

125		Egea Molinas, M.T. Etnobotanica nell'Alta Valle del Reno (Toscana ed Emilia-Romagna, Italia). PhD Thesis, Universidad Miguel Hernández de Elche, Orihuela, 22 february 2016, pp. 524.	Emilia-Romagna, Tuscany
126		Fagioli, L.; Marini, D. Piante alimentari ed officinali di Forni di Sopra nel Parco Naturale delle Dolomiti Friulane. Ed. Parco Naturale Dolomiti Friulane, 2006; pp. 85.	Friuli Venezia Giulia
127		Fenaroli, L. Florae Garganicae Mantissa *Delpinoa* **1972-1973,** n.s., 14-15.	Apulia
128		Ficarra, P.; Scaccabarozzi S. Buoni da mangiare. Erbe e frutti selvatici delle vallate dei Nebrodi; 2011; pp. 293 http://piantespontaneeincucina.info/wordpress/?page_it=58.	Sicily
129		Fioretti, E. *L'arca delle verdure (Progetto n° 4). Relazione dettagliata sull'attività svolta e sui risultati ottenuti nel terzo anno di attuazione del progetto e relazione finale* Dipartimento di Biologia MCA, Università di Camerino 2010.	Marche
130		Fossati, F.; Bianchi, A.; Favali, M. A. Farmacopea popolare del Parmense: passato e presente. *Inf. Bot. Ital.* **1999,** *31* (1-3), 171-176.	Emilia Romagna
131		Galt, A.H.; Galt, J.W.; Peasant use of some wild plants on the Island of Pantelleria, Sicilia. *Economic Botany* **1978,** *32* , 20-26.	Sicily
132		Gargano, M.L.; Venturella, G.; Lazzara, S.; Lo Nardo, R.; Saporita, P. Ethnobotanical knowledge in some rural communities of northern Sicily (Palermo, Italy). *Atti Soc.Tosc. Sci. Nat., Mem.* **2018**, Serie B, *125* , 31-40.	Sicily
133		Gastaldo, P.; Barberis, G.; Fossati, F. Le piante della medicina tradizionale nei dintorni di Praglia (Appennino Ligure-Piemontese). *Atti Acc. Lig. Sci. Lett. (Genova)* **1978**, *35,* 1-35.	Liguria
134		Geraci, A.; Amato, F.; Di Noto, G.; Bazan, G.; Schicchi, R. The wild taxa utilized as vegetables in Sicily (Italy): a traditional component of the Mediterranean diet. *J. Ethnobiol. Ethnomed.* **2018,** *14* :14.	Sicily
135		Ghirardini, M.P.; Carli, M.; del Vecchio, N.; Rovati, A.; Cova, O.; Valigi, F.; Agnetti, G.; Macconi, M.; Adamo, D.; Traina, M.; Laudini, F.; Marcheselli, I.; Caruso, N.; Gedda, T.; Donati, F.; Marzadro, A.; Russi, P.; Spaggiari, C.; Bianco, M.; Binda, R.; Barattieri, E.; Tognacci, A.; Girardo, M.; Vaschetti, L.; Caprino, P.; Sesti, E.; Andreozzi, G.; Coletto, E.; Belzer, G.; Pieroni, A. The importance of a taste a comparative study on wild food plant consumption in twenty-one local communities in Italy. *J. Ethnobiol. Ethnomed* . **2007**, *3* (22), 1-14.	Italy
136		Giachetti, G.,Tomei P. E. Piante officinali nell'uso tradizionale del territorio mugellano (Toscana). *Atti Soc. It. Sci. nat. Museo civ. Stor. nat. Milano* **2003**, *144* (1), 23-49.	Tuscany
137		Giannotti, G. *Piante spontanee commestibili Comunità Montana Cigno Valle Biferno Casacalenda (CB);* 2008; pp. 76.	Molise
138	*	Giardino Botanico Lorenzo Rota di Bergamo. *Le nostre erbe: piante alimentari spontanee;* Ed. Junior: Bergamo, Italy, 1996; pp. 120.	Lombardia
139		Giusti, M.E.; Nebel, S.; Pieroni, A. Erbe e percezione del sapore tra gli Arbëreshë del Vulture in Lucania. *La Ricerca Folklorica* **2002,** *45* , 29–41.	Basilicata
140		Giusti, M.E.; Pieroni, A. Cercare, raccogliere ed utilizzare piante spontanee (e non). Alcune indagini etnoscientifiche in Provincia di Lucca. *Boll. Acc. Euteleti della Città di San Miniato (Italy)* **2009**, *76,* 429-460.	Tuscany
141		Guarino, C.; De Simone, L.; Santoro, S. Ethnobotanical Study of the Sannio Area, Campania, Southern Italy. *Ethnobotany Research Applications* **2008**, *6,* 255-317.	Campania
142		Guarrera, P.M. Food Medicine and Minor Nourishment in the Folk Traditions of Central Italy (Marche, Abruzzo and Latium). *Fitoterapia* **2003**, *74* (6), 515–544.	Marche, Abruzzi, Latium
143		Guarrera, P.M. Il patrimonio etnobotanico del Lazio. Le piante del Lazio nell'uso terapeutico, alimentare, domestico, religioso e magico; Regione Lazio, Assessorato alla Cultura e Dipartimento di Biologia Vegetale Università "La Sapienza", Tip. Tipar: Roma, Italy, **1994**.	Latium
144		Guarrera, P.M. Le piante nelle tradizioni popolari del Lazio. *Erboristeria Domani* **2004,** 75-84.	Latium
145		Guarrera, P.M. Le piante nelle tradizioni popolari del Molise. *Erboristeria Domani* **2009,** 335, 58–64.	Molise
146		Guarrera, P.M. Le piante nelle tradizioni popolari dell'Emilia-Romagna. *Erboristeria Domani* **2008,** 329, 58–69.	Emilia Romagna
147		Guarrera, P.M. Le piante nelle tradizioni popolari della Basilicata. *Erboristeria Domani* **2005,** 56–63.	Basilicata
148		Guarrera, P.M. Le piante nelle tradizioni popolari della Liguria. *Erboristeria Domani* **2008,** *319* , 61-67.	Liguria

149	Guarrera, P.M. Le piante nelle tradizioni popolari della Lombardia. *Erboristeria Domani* **2008,** *30* , 60–69.	Lombardia
150	Guarrera, P.M. Le piante nelle tradizioni popolari dell'Abruzzo. *Erboristeria Domani* **2004,** 53-60.	Abruzzi
151	Guarrera, P.M. Le piante nelle tradizioni popolari delle Marche. *Erboristeria Domani* **2005,** 52-59.	Marche
152	Guarrera, P.M. Primo contributo allo studio del patrimonio etnobotanico del Molise Atti del 97° Congresso della Società Botanica Italiana, Lecce, 24-27 settembre 2002, p. 209.	Molise
153	Guarrera, P.M. Ricerche etnobotaniche nelle province di Macerata e di Ancona. *EPPOS* **1981,** *63* (2), 99–108, *63* (4), 220–228.	Marche
154	Guarrera, P.M. Usi e Tradizioni della Flora Italiana. Medicina, Popolare ed Etnobotanica. Ed. Aracne: Roma, Italy, 2006; pp. 436.	Italy
155	Guarrera, P.M. Usi Tradizionali delle piante in alcune aree marchigiane. *Inf. Bot. Ital.* **1990,** *22* (3), 155-167.	Marche
156	Guarrera, P.M. *Usi tradizionali delle piante nel territorio della Majella. Rivista Abruzzese, Fascic. Monograf. su Erbe e Piante Medicinali nella Storia e nelle Tradizioni Popolari Abruzzesi;* Centro Servizi Culturali Regione Abruzzo, Off. Grafiche Anxanum, Lanciano, Italy, 1987**;** pp. 17-44.	Abruzzi
157	Guarrera, P.M.; Castellacci, A.M.; Tacconi, M. Gli usi tradizionali delle piante (nella zona dei Castelli romani). *Agricoltura Ambiente* **1984**, *24,* 46-64.	Latium
158	Guarrera, P.M.; Forti, G.; Marignoli, S. Ethnobotanical and ethnomedicinal uses of plants in the district of Acquapendente (Latium, Central Italy) *J. Ethnopharmacol.* **2005**, *96* , 429-444.	Latium
159	Guarrera, P.M.; Forti, G.; Marignoli, S.; Gelsomini, G. Piante e tradizione popolare ad Acquapendente. *Quaderni del Museo del Fiore n.2.* Comune di Acquapendente, regione Lazio. Acquapendente, 2004.	Latium
160	Guarrera, P.M.; Leporatti, M. Ethnobotanical remarks on Central and Southern Italy. *J. Ethnobiol. Ethnomed.* **2007**, *3* , 23-33.	Central and Southern Italy
161	Guarrera, P.M.; Lucchese, F.; Medori, S. Ethnophytotherapeutical research in the high Molise region (Central-Southern Italy). *J. Ethnobiol. Ethnomed.* **2008,** *4* (1), 1-11.	Molise
162	Guarrera, P.M.; Lucchese, F.; Medori, S. *L'uso tradizionale delle piante nell'Alto Molise.* Ed. Tipar arti Grafiche: Firenze, Italy, 2009; pp. 127.	Molise
163	Guarrera, P.M.; Manzi A. Wild plants of organoleptic or nutritional interest and food traditions in central Italy: some in teresting cases. *Plant Genet.Resour.* **2005,** *3* (3), 322–325.	Central Italy
164	Guarrera, P.M.; Nicoletti, M. I nutraceutici,o alimenti-medicina,e la tradizione popolare. In *Etnobotanica. Conservazione di un patrimonio culturale come risorsa per uno sviluppo sostenibile;* Caneva, G., Pieroni, A., Guarrera, P.M., Eds.; Edipuglia: Santo Spirito (Ba), Italy, 2013; pp. 289–293.	Italy
165	Guarrera, P.M.; Salerno, G.; Caneva, G. Food, flavouring and feed plant traditions in the Tyrrhenian sector of Basilicata, Italy. *J. Ethnobiol. Ethnomed.* **2006,** *2* (1), 1-6.	Basilicata
166	Guarrera, P.M.; Salerno, G.; Caneva, G. Indagini etnobotaniche nel versante tirrenico della Basilicata. Atti del 98° Congresso della Società Botanica Italiana, Catania, 24-26 settembre 2003**,** p. 138.	Basilicata
167	Guarrera, P.M.; Salerno, G.; Caneva, G. Le tradizioni etno-e farmacobotaniche del territorio di Maratea. In *Flora, vegetazione e tradizioni etnobotaniche di Maratea;* Cutini, M., Caneva, G., Eds.; Gangemi Ed.: Rome, Italy, 2009; pp. 141–168.	Basilicata
168	Guarrera, P.M.; Savo, V. Perceived health properties of wild and cultivated food plants in local and popular traditions of Italy: a review. *J. Ethnopharmacol.* **2013**, *146* (3), 659–680.	Italy
169	Guarrera, P.M.; Savo, V. Wild food plants used in traditional vegetable mixtures in Italy. *J. Ethnopharmacology* **2016,** *185* , 202–234.	Italy
170	Guarrera, P.M.; Tammaro, F. Piante amaro-aromatiche utilizzate nell'alimentazione ed in liquoristica in Abruzzo e in altre zone d'Italia. Atti del Convegno Internazionale "Funghi, tartufi ed erbe mangerecce". L'Aquila, 28 settembre-1 ottobre 1995; pp.105–119.	Abruzzi
171	Guerci, A. La ricerca e la didattica etonobotanica in Italia: stato attuale e prospettive. *Inf. Bot. Ital.* **1999,** *31* (1-3), 99 -102.	Italy

172	Hadjichambis, A.C.; Paraskeva-Hadjichambi, D.; Della, A.; Giusti, M.E.; De Pasquale, C.; Lenzarini, C.; Censorii, E.; Gonzales-Tejero, M. R.; Sanchez-Rojas, C. P.; Ramiro-Gutierrez, J. M.; Skoula, M.; Johnson, C.; Sarpaki, A.; Hmamouchi, M.; Jorhi, S.; El-Demerdash, M.; El-Zayat, M.; Pieroni, A. Wild and semidomesticated food plant consumption in seven circum-Mediterranean areas. *Int. Journal of Food Sciences and Nutrition* **2008**, *59* , 383-414	Mediterranean
173	Hammer, K.; Laghetti, G.; Cifarelli, S.; Spahillari, M.; Perrino, P. Pimpinella anisoides Briganti. *Genetic Resources and Crop Evolution* **2000**, *47* , 223–225.	Basilicata
174	Idolo, M.; Motti, R.; Mazzoleni, S. Ethnobotanical and phytomedicinal knowledge in a long-history protected area, the Abruzzo, Lazio and Molise National Park (Italian Apennines). *J. Ethnopharmacol.* **2010,** *127* , 379–395.	Abruzzi
175	Iuliano, L.; Grotteria, M.; Pascoli, D.; Rispoli, P.; Sigilli, A.; Sirianni, R.M. *Le piante della tradizione calabrese;* ARSSA Calabria, 2002; pp.125. http://www.arsacweb.it/wpcontent/uploads/2017/07/Le-piante-della-tradizione-Calabrese_ARSAC_1.pdf	Calabria
176	La Rocca, A.; Cornara, L.; Mariotti, M.G. Fitoalimurgia nella riviera ligure di levante. Atti 103° Congresso S.B.I., Reggio Calabria. 17-19.9.2008, p. 146.	Liguria
177	La Rocca, A.; Mariotti, M.G.; Cornara, L. Piante spontanee di interesse alimentare in Liguria. Atti 105° Congresso Nazionale S.B.I.,Univ.di Milano. 25-28/8/2010, 2010; p. 109.	Liguria
178	La Rocca, A.; Cornara, L.; Mariotti, M.G.; Terrizzano, L. *Piante e tradizioni a Cosio d'Arroscia Boves (CN);* Ed. Araba Fenice, 2012; pp. 160.	Liguria
179	Laghetti, G.; Pignone, D.; De Lisi, A.; Cifarelli, S.; Faslia, N.; Hammer, K. Collecting crop genetic resources in Italian towns of Albanian origin across the Molise, Calabria and Sicily regions. *Genetic Resources and Crop Evolution* **2011**, *58* (1), 139–152.	Molise, Calabria, Sicily
180	Lancioni, M.C.; Ballero, M.; Mura, L.; Maxia, A. Usi alimentari e terapeutici nella tradizione popolare del Goceano (Sardegna centrale). *Atti Soc. tosc. Sci. nat., Mem.* **2007**, Serie B, *114* , 45-56.	Sardinia
181	Lanzan, L.A. La medicina popolare della Valsesia. In *Herbora,* Atti delle giornate 23-28 maggio 1979; pp. 338-341.	Piemonte
182	Lentini, F. Gli usi tradizionali delle piante di Sant'Angelo Muxaro. In Atti del Convegno" *Natura, Mito & Storia nel Regno Sicano di Kokalos* " Sant'Angelo Muxaro (Agrigento) **1996,** 33-39.	Sicily
183	Lentini, F. Indagini etnobotaniche in Sicilia. II. L'uso tradizionale delle piante in alcune comunità del Trapanese. Atti del Convegno "Ricerca Sperimentale in Farmacobotanica" (Urbino, 25-26 Settembre 1987), "Studi Urbinati" **1987,** *LX* , s. C, *29* , 151-167.	Sicily
184	Lentini, F. L'etnobotanica in Sicilia: le piante alimentari di uso popolare. In *Etnobotanica nella Provincia di Catania con Atti del Convegno "Andar per verdure";* Nuova Zangara Stampa Editrice: Linguaglossa, Italy, 2002.	Sicily
185	Lentini, F. The role of ethnobotanics in scientific research. State of ethnobotanical knowledge in Sicily. *Fitoterapia* **2000,** *71* , 83-88.	Sicily
186	Lentini, F.; Aleo, M.; Amenta, R. L'uso popolare delle piante nelle isole Egadi (Sicilia). *Acta Phytoterapeutica* **1997,** *4* (2): 88-94.	Sicily
187	Lentini, F.; Aleo, M.; Amenta, R. L'uso popolare delle piante nelle isole Egadi (Sicilia). *Giorn. Bot. Ital.* **1993,** *127* (3), 702.	Sicily
188	Lentini, F.; Amenta R. Indagini sul'uso tradizionale delle piante della flora locale nelle comunità dell'Agrigentino. *Giorn. Bot. Ital.* **1992,** 126 (2): 371.	Sicily
189	Lentini, F.; Catanzaro, F.; Aleo, M. Indagine etnobotaniche in Sicilia. III. L'uso tradizionale delle piante nel territorio di Mazzara del Vallo (Trapani). *Atti dell'Accademia di Scienze Lettere e Arti di Palermo* **1988,** 1-29.	Sicily
190	Lentini, F.; Di Martino, A.; Amenta, R. Contributo alla conoscenza della flora popolare dell'isola di Ustica. *Quad. Bot. Amb. Appl. Palermo* **1994**, *5* , 47-54.	Sicily
191	Lentini, F.; Di Martino, A.; Amenta, R. La flora popolare di Ustica (Palermo). *Giorn. Bot. Ital.* **1996,** *129* (2), 167.	Sicily
192	Lentini, F.; Giani, S.; Amenta, R. L'uso popolare delle piante nelle isole Eolie (Sicilia). Acta technologiae et legis medicamenti. *Pharmacia mediterranea* **1995**, *6* (3): 351-355.	Sicily
193	Lentini, F.; Raimondo, F. M. Indagini etnobotaniche in Sicilia. IV. L'uso tradizionale delle piante nel territorio di Mistretta (Messina). *Quad. Bot. Amb. Appl* . **1990,** *1* , 103-117.	Sicily

194		Lentini, F.; Venturella, G.; Raimondo, F. M. Sintesi delle indagini etnobotaniche condotte in Sicilia. *Giorn. Bot. Ital.* **1988,** *122* (1), 221.	Sicily
195		Lentini, F.; Venza F. Valorizzazione delle piante selvatiche di uso alimentare in Sicilia. Atti 2° Convegno Nazionale Piante Mediterranee, Agrigento, 8.10.2004.	Sicily
196		Lentini, F.; Venza, F. Wild food plants of popular use in Sicily. *J. Ethnobiol. Ethnomed.* **2007,** *3* (1), 15.	Sicily
197		Lentini, F; Aleo, M. Indagini etnobotaniche in Sicilia. V. L'uso tradizionale delle piante nel territorio di Erice (Trapani). *Atti Accad. Sci. Lett. Arti Palermo* **1991,** *XI* , 67-98.	Sicily
198	*	Lentini, F; Di Martino, A; Amenta, R. La flora popolare dell'Isola di Pantelleria (TP). In VIII Convegno S.I.F. "*Alla scoperta della Digitale del Gennargentu* ", Arzana (Nuoro), 1996, 70.	Sicily
199		Lentini, F; Di Martino, A; Amenta, R. Le piante di uso popolare nell'arcipelago delle Pelagie (AG). *L'uomo e l'ambiente* **1995**, *19,* 117-121.	Sicily
200		Leonti, M.; Nebel, S.; Rivera, D.; Heinrich, M. Wild gathered food plants in the European Mediterranean: A comparative analysis. *Economic Botany* **2006,** *60* (2), 130–142.	Basilicata, Calabria
201		Leporatti, M. L.; Guarrera, P. M. Contributo alla conoscenza degli usi tradizionali delle piante in Capitanata e Salento (Puglia). Atti del 99° Congresso della Società Botanica italiana, Torino, 22-24 settembre 2004, pp. 285.	Apulia
202		Leporatti, M.L.; Corradi, L. Ethnopharmacobotanical remarks on the Province of Chieti town (Abruzzo, Central Italy). *J. Ethnopharmacol.* **2001,** *74* , 17–40.	Abruzzi
203		Leporatti, M.L.; Guarrera, P.M. Ethnobotanical remarks in Capitanata and Salento areas (Puglia, Southern Italy). *Etnobiology* **2007,** *5,* 51–64.	Apulia
204		Leto, C.; Tuttolomondo, T.; La Bella, S.; Bonsanguea G.; Venturella G.; Gargano, M.L.; Savo, V.; Licata, M. Ethnobotanical investigation on wild plants in the Monti Sicani Regional Park (Sicily, Italy). *Journal of Ethnopharmacology,* **2014,** *153 (* 3), 568-586.	Sicily
205		Licata, M.; Tuttolomondo, T.; Leto, C.; Virga, G.; Bonsangue, G.; Cammalleri, I.; Gennaro, M.C.; La Bella, S. A survey of wild plant species for food use in Sicily (Italy) – results of a 3-year study in four Regional Parks. *J. Ethnobiol. Ethnomed* . **2016,** *12* :12.	Sicily
206		Lippi, A. *Da erbe per i poveri a cibo per i buongustai: la riscoperta delle piante selvatiche di uso alimentare* . Ed. Ponte, Lucca, Italy, 1998; pp. 31.	Tuscany
207		Lippi, S. L'uso delle piante nella tradizione popolare del territorio comunale di Massarosa (LU) (Botrici, Camisano, Casesi, Mommio Castello, I Venti). *Paralleli e Meridiani* **2005,** Anno III, N. *1* , 7-44.	Tuscany
208		Lomagno Caramiello, R.; Piervittori, A.; Lomagno, P.A.; Rolando C. Fitoterapia popolare nelle Valli Chisoni e Germanasca. *Ann. Fac. Sci. Agr. Univ. Torino* **1984,** *XIII* , 259-298.	Piemonte
209		Lomagno, P.; Lomagno Caramiello, R. La fitoterapia popolare nella valle di Susa. *Allionia* **1970,** *16* , 165-174.	Piemonte
210		Longhi, M.; Scanavino, C. Alpi occidentali. Una ricerca sulle tradizioni erboristiche. *Erboristeria domani* **1986,** *11* , 54-58.	Piemonte
211		Lucchetti, L.; Zitti, S.; Taffetani, F. Ethnobotanical uses in the Ancona district (Marche region, Central Italy). *J. Ethnobiol. Ethnomed.* **2019,** *15* (1), 1-33.	Marche
212		Lupia, A.; Lupia, C.; Lupia R. *Etnobotanica in Calabria.Viaggio alla scoperta di antichi saperi intorno al mondo delle piante.* Ed. Rubbettino: Soveria Mannelli (CZ), Italy, 2017; pp. 341.	Calabria
213		Lupia, C. Etnobotanica. *Le piante e i frutti spontanei nella Sila catanzarese.* Ed. Abramo, Catanzaro, Italy, 2004; pp. 99.	Calabria
214		Lupia, C.; Lupia, R. *Etnobotanica: piante e tradizioni popolari di Calabria.* Congi: Crotone, Italy, 2014.	Calabria
215		Macchi, M. P. Schede etnobotaniche delle piante alimurgiche dell'Alto Garda 2017 https://www.judicaria.it/wp-content/uploads/2019/04/schede-piante-alimurgiche-Alto-Garda-2017.pdf	Trentino Alto-Adige
216		Maccioni, S.; Flamini, G.; Cioni, P.L.; Bedini, G.; Guazzi, E. Ricerche etnobotaniche in Liguria. La riviera spezzina (Liguria orientale). *Atti Soc. tosc. Sci. nat., Mem.* **2008**, Serie B, *115,* 77-82.	Liguria
217		Maccioni, S.; Monti, G.; Flamini, G.; Cioni, P.L.; Morelli, I.; Guazzi, E. Ricerche etnobotaniche in Liguria. La Val Lerrone e la bassa Valle Arroscia. *Atti Soc. tosc. Sci. Nat. Mem* . **2005,** Serie B, *111* (2004), 129-134.	Liguria

218		Maccioni, S.; Tomei, P. E.; Rizzo, A. L'uso medicinale delle specie vegetali selvatiche e coltivate nella tradizione popolare della bassa Val di Magra. *Mem. Acc. Lunigianese Sci., "G. Cappellini"* **1994-1995**, *LXIV-LXV*, 389-435.	Tuscany
219		Maggi F. *Piante officinali dell'alto maceratese: usi e tradizioni popolari tra passato e presente. Proposte e Ricerche* **2006,** *56*, 122-145.	Marche
220		Mambrini, M.; Vicarelli, G.B. *Piante officinali dell'Amiata. Usi e tradizioni popolari Castell'Azzara (Grosseto);* Cooperativa Agricola Forestale dei Comuni Amiatini, 1983.	Tuscany
221		Mancini, E.; De Martino, L.; Malova, H.; De Feo, V. Chemical composition and biological activities of the essential oil from Calamintha nepeta plants from the wild in southern Italy. *Nat. Prod. Commun*. **2013**, *8* (1), 139–142.	Southern Italy
222		Manzi, A. *I mugnoli di Pettorano sul Gizio. La gustosa verdura degli ortolani-pastori.* Riserva Naturale Regionale Monte Genzana-Alto Gizio 2015; pp. 32.	Abruzzi
223		Manzi, A. La cultura dei frassini da manna in Abruzzo. *Rivista Abruzzese* **1989,** *4*, 355-357.	Abruzzi
224		Manzi, A. *Le piante alimentari in Abruzzo;* Ed. Tinari: Villamagna (Chieti), Italy, 1999.	Abruzzi
225		Manzi, A. Piante spontanee utilizzate nell'alimentazione umana nel territorio di Gessopalena (Abruzzo). *Inform. Bot. Ital*. **1987,** *19*, 257-264.	Abruzzi
226	*	Marinoni, J. *Cucina e salute con le erbe spontanee nelle Tre Venezie.* Padova, 1984.	Veneto
227		Marra, L. *Monte Olacio;* Ed. Libreria Colacchi: L'Aquila, Italy, 2005; pp. 131.	Abruzzi
228	*	Marsano, M. *Le piante officinali e i prodotti del sottobosco nel Goriziano*; Ed. Tip. L. Lucchesi, 1941.	Friuli Venezia Giulia
229		Martelli, I. Braca A., Camangi F. Tradizioni etnofarmacobotaniche nel territorio del Gabbro (Livorno-Toscana). *Quad. Mus. St. Nat. Livorno* **2015,** 26, 15-38.	Tuscany
230		Martini, E. La Fitoterapia Popolare in Val Borbera (Appennino Ligure). *Webbia* **1982,** 35(1), 187-205.	Piemonte
231		Martini, E. La Fitoterapia Popolare nell'alta Valle dell' Orba (Appennino Ligure). *Atti Acc. Ligure Sc. Lett.* (Genova) **1982,** 39, 1-25.	Liguria
232		Maruca, G.; Spampinato, G.; Turiano, D.; Laghetti, G.; Musarella, C. M. Ethnobotanical notes about medicinal and useful plants of the Reventino Massif tradition (Calabria region, Southern Italy). *Genet Resour Crop Evol* **2019,** *66*, 1027–1040.	Calabria
233		Mattalia, G.; Cassandra, L.; Quave, C.L.; Pieroni, A. Traditional uses of wild food and medicinal plants among Brigasc, Kyé, and Provençal communities on the Western Italian Alps. *Genet. Resour. Crop Evol.* **2013**, 60 (2): 115-132.	Northern Italy (western Alps)
234		Mattalia, G.; Corvo, P.; Pieroni, A. The virtues of being peripheral, recreational, and transnational: local wild food and medicinal plant knowledge in selected remote municipalities of Calabria, Southern Italy. *Ethnobotany Research & Applications* **2020,** 19: 1-20.	Calabria
235		Mattalia, G.; Sõukand, R.; Corvo, P.; Pieroni, A. "We Became Rich and We Lost Everything": Ethnobotany of Remote Mountain Villages of Abruzzo and Molise, Central Italy. *Human Ecology*, **2021**, 1-8.	Abruzzi, Molise
236		Mattalia, G.; Sõukand, R.; Corvo, P.; Pieroni, A. Blended divergences: local food and medicinal plant uses among Arbëreshë, Occitans, and autochthonous Calabrians living in Calabria, Southern Italy. *Plant Biosystems* **2020,** *154* (5), 615-626.	Calabria
237		Mattalia, G.; Sõukand, R.; Corvo, P.; Pieroni, A. Dissymmetry at the Border: Wild Food and Medicinal Ethnobotany of Slovenes and Friulians in NE Italy. *Economic Botany* **2020**, 1-14.	Friuli Venezia Giulia
238		Mattirolo, O. Phytoalimurgia Pedemontana (ossia Censimento delle specie vegetali alimentari della flora spontanea del Piemonte). *Annali della R. Accademia d'agricoltura di Torino* **1918**, *61*.	Italy
239		Mattirolo, O.; Gallino, B.; Pallavicini, G. *Phytoalimurgia pedemontana. Come alimentarsi con le piante selvatiche*; Ed. Blu Edizioni: Torino, 2017; pp. 335.	Piemonte
240		Mautone, M.; De Martino, L.; De Feo, V. Ethnobotanical research in Cava de' Tirreni area, Southern Italy. *J. Ethnobiol. Ethnomed*. **2019,** *15* (1): 1-21.	Campania
241		Mazzola, P.; Nibali Lupica, S.; Bartolotta, L. Contributo alla conoscenza delle piante alimurgiche del Parco dei Nebrodi: le specie utilizzate nella tradizione popolare di Caronia (Messina). *Quad. Bot. Amb. Appl*. **2015,** *26*, 3-10.	Sicily
242		Mearelli, F.; Tardelli, C. Maremma mediterranea. *Erboristeria domani* **1995,** 45-57.	Tuscany
243		Medagli, P.; Accogli, R.; Turco, A.; Zuccarello, V.; Albano, A. Fiori spontanei del Salento. Guida al riconoscimento e alla tutela; Edizioni del Grifo, 2016; pp. 144.	Apulia

244		Menale, B.; Amato, G.; Di Prisco, C.; Muoio, R. Traditional uses of plants in North-Western Molise (Central Italy). *Delpinoa* **2006,** *48*, 29–36.	Molise
245	*	Mezzena, R.T. *Frutti selvatici, mangerecci e velenosi del Carso.* Trieste, Fondazione Cassa di Risparmio di Trieste, 2002.	Friuli Venezia Giulia
246		Molinaro, F. *Menesta asciatizza. A tavola con le piante spontanee dell'Appennino meridionale;* Ed. La bancarella: Piombino, Italy, 2011.	Southern Italy
247		Montesano, V.; Negro, D.; Sarli, G.; De Lisi, A.; Laghetti, G.; Hammer, K. Notes about the uses of plants by one of the last healers in the Basilicata Region (South Italy). *J. Ethnobiol. Ethnomed.* **2012,** 8:15.	Basilicata
248		Morreale, F. Piante Spontanee Alimentari in Sicilia. Guida di fitoalimurgia; Ed. Casa Editrice Natura Sicula, 2018; pp. 319.	Sicily
249		Motti, R.; Antignani, V.; Idolo, M. Traditional Plant Use in the Phlegraean Fields Regional Park (Campania, Southern Italy). *Hum. Ecol.* **2009,** *37,* 775–782.	Campania
250		Motti, R.; Bonanomi, G.; Lanzotti, V.; Sacchi, R. The Contribution of Wild Edible Plants to the Mediterranean Diet: An Ethnobotanical Case Study Along the Coast of Campania (Southern Italy). *Economic Botany*, **2020,** 74(3), 249-272.	Campania
251		Motti, R.; Motti, P. An Ethnobotanical Survey of Useful Plants in the Agro Nocerino Sarnese (Campania, Southern Italy). *Hum. Ecol.* **2017,** *45*, 865–878.	Campania
252		Musarella, C.M.; Paglianiti, I.; Cano-Ortiz, A.; Spampinato, G. Indagine etnobotanica nel territorio del Poro e delle Preserre Calabresi (Vibo Valentia, S-Italia). *Atti Soc. Tosc. Sci. Nat., Mem*., Serie B, **2019,** *126*, 13-28.	Calabria
253		Napoli, M. Usi popolari di Barlia robertiana (Loisel.) W. Greut. (Orchidaceae) nel territorio di Santo Pietro di Caltagirone (Catania). In *Etnobotanica nella Provincia di Catania con Atti del Convegno "Andar per verdure";* Nuova Zangara Stampa Editrice: Linguaglossa, Italy, 2002.	Sicily
254		Nardelli, G. M. *Cultura e tradizione. Demomedicina nell'alta Umbria.* Provincia di Perugia. 1987.	Umbria
255		Nardone, D. Piante e frutti spontanei nel Salento. In *Etnobotanica nella Provincia di Catania con Atti del Convegno "Andar per verdure";* Nuova Zangara Stampa Editrice: Linguaglossa, Italy, 2002.	Apulia
256		Navoni, G.; Vietina, B.; Bracelli, F.; Ardara, N. *Erbe spontanee commestibili nella tradizione massese;* Ceccotti Ed.: Massa, Italy, 1997.	Tuscany
257		Nebel, S.; Heinrich, M. Ta chòrta: A comparative ethnobotanical-linguistic study of wild food plants in a graecanic area in Calabria, Southern Italy. *Economic Botany*, **2009,** *63* (1), 78-92.	Calabria
258		Nebel, S.; Heinrich, M. The use of wild edible plants in the Graecanic area in Calabria, Southern Italy. *Ethnobotany of the New Europe*, **2010,** 172-188.	Calabria
259		Nebel, S.; Pieroni, A.; Heinrich, M. Ta Chorta: Wild edible greens used in the Graecanic area in Calabria, Southern Italy. *Appetite*, **2006,** 47(3), 333–342.	Calabria
260		Ossato, R.; Chiesura, F.; Scortegagna, S. Ricerche etnobotaniche nel territorio vicentino: utilizzazione di piante spontanee e coltivate nella Valle di Laghi (Prealpi Vicentine – Veneto). *Natura Vicentina* **2007,** 11: 145-158.	Veneto
261		Pallabazzer, V. Infanzia, Natura ed economia nelle valli dolomitiche fino alla seconda guerra mondiale. *Atti Ac. Rov. Agiati* **1997,** VII (7): 141-150.	Veneto
262		Palmese, M. T.; Uncini Manganelli, R. E.; Tomei, P. E. An ethnoparmacobotanical survey in the Sarrabus district (South-east Sardinia). *Fitoterapia* **2001,** *72*, 619-643.	Sardinia
263		Paoletti, M. G.; Dreon, A. L.; Lorenzoni, G.G. Pistic, Traditional Food from Western Friuli, N.E. *Italy Economic Botany* **1995,** *49*, 26-30.	Friuli Venezia Giulia
264		Pasta S, Garfì G, La Bella F, Rühl J, Carimi F. An overview on the human exploitation of Sicilian native edible plants. In: Davis ER, editor. *Wild plants: identification, uses and conservation*. Nova Science Publishers Press: Hauppauge, NY, 2011; pp. 1–74.	Basilicata
265		Pasta, S.; La Rosa, A.; Garfì, G.; Marcenò, C.; Gristina, A.S.; Carimi, F.; Guarino, R. An Updated Checklist of the Sicilian Native Edible Plants: Preserving the Traditional Ecological Knowledge of Century-Old Agro-Pastoral Landscapes. *Frontiers in Plant Science* **2020,** *11:* 388.	Sicily
266	*	Pedrotti, G., Bellomaria, B. *Piante officinali spontanee delle Marche;* Atti I Convegno Naz. Erboristeria e Piante Officinali. Recanati **1978,** pp. 71-74.	Marche
267		Pedrotti, G.; Bertoldi, V. *Nomi dialettali delle piante indigene del Trentino e della Ladinia dolomitica presi in esame dal punto di vista della botanica della linguistica e del folklore.* Ed. G. Monauni, Trento, 1930.	Trentino Alto-Adige

268	Perno, L.; Corsi, G.; Miraldi, E. Aspetti etnobotanici nel territorio di Rio dell'Elba. *Atti Soc. toss. Sci. nat., Mem.* , Serie 8, **1997,** *104* , 43-51.	Tuscany
269	Perrino, E.V.; Signorile, G. Indagini etnobotaniche in alcune località della Puglia Centrale. *Boll. Mus. Ist. Biol. Univ. Genova* , **2011**, 73.	Apulia
270	Perrone, C.; Medagli, P. Le forze della natura. Le Piante nel territorio gallipolino tra Storia, Mitologia e Folklore. Coordinamento editoriale Centro Regionale Servizi Educativi e Culturali C.R.S.E.C. LE/48 - Gallipoli 2006.	Apulia
271 *	Persico, G.; Carra, G. *Erbe in tavola. Piante spontanee commestibili della Pianura Padana. Note botaniche e di cucina;* Ed. Tre Lune, 2003.	Italy
272	Pezzotta, R. *Saperi naturalistici locali;* Centro Educazione Ambientale di Cerreto di Spoleto, Centro per la Documentazione e la Ricerca Antropologica in Valnerina. Centro Stampa Regionale: Perugia, Italy, 1994.	Umbria
273	Picchi G., Pieroni A. *Atlante dei prodotti tipici* . Le erbe; Istituto Nazionale di Sociologia Rurale, RAI, Agraed.: Roma, Italy, 2005.	Italy
274	Picchi, G.*Terra e cibo della Marca d'Ancona.* Provincia di Ancona, Assessorato alle Politiche Agricole: Ancona, Italy, 2002.	Marche
275	Pieroni, A. "Erbi boni" nelle tradizioni gastronomiche garfagnine. In *Erbi boni, erbi degli streghi/ Good weeds, witches'weed* s. Atti del Seminario di Studio, Gallicano (LU), maggio 1997: Pieroni, A., Ed; Experiences Verlag: Köln, Germany, 1998; pp. 3-18.	Tuscany
276	Pieroni, A. *Etnobotanica a Castelmezzano. Manuale sull'uso delle erbe officinali in un borgo della Lucania, Accettura, Italy.* Ed. Le Macine 2007	Basilicata
277	Pieroni, A. Evaluation of the cultural significance of wild food botanicals traditionally consumed in Northwestern Tuscany, Italy. *Journal of Ethobiology* **2001,** *21* (1), 89-104	Tuscany
278	Pieroni, A. Gathered wild food plants in the upper valley of the Serchio river (Garfagnana), Central Italy. *Economic botany* **1999,** *53* (3), 327-341.	Tuscany
279	Pieroni, A. Medicinal plants and food medicines in the folk traditions of the upper Lucca Province, Italy. *J. Ethnopharmacology* **2000**, *70,* 235–273.	Tuscany
280	Pieroni, A. Piante spontanee della tradizione ed immaginario collettivo in Alta Garfagnana (Lucca): un centro di documentazione sulla cultura orale. *Inf. Bot. Italiano* **1999,** *31* (1-3), 183-189.	Tuscany
281	Pieroni, A. Toxic plants as food plants in the traditional uses of the Eastern Apuan Alps Region, North-West Tuscany, Italy. In *Il cibo ed il corpo. Dal cibo alla cultura, dalla cultura il cibo* ; Guerci, A., Ed; Erga ed.: Genova, Italy, 1999; pp. 262-272.	Tuscany
282	Pieroni, A. Wild food plants and Arbëresh women in Lucania, Southern Italy. In *Women and plants: Gender relations in biodiversity management and conservation* ; Howard, P.L., Ed.; The University of Chicago Press.: Chicago, 2003; pp. 66–82.	Basilicata
283	Pieroni, A.; Giusti, M.E. Alpine ethnobotany in Italy: traditional knowledge of gastronomic and medicinal plants among the Occitans of the upper Varaita valley, Piedmont. *J. Ethnobiol. Ethnomed.* **2009,** *5* :32	Piemonte
284	Pieroni, A.; Giusti, M.E. Spazio e paesaggio nell'immaginario legato alle erbe delle comunità Arbreshe del Vulture in Lucania. In *Tradizioni Popolari.* Atti del Convegno "Immaginario,Territorio, Paesaggio", Piazza al Serchio (Lucca), 9 Dicembre 2000; Experieces Verlag: Koln, **2002**: 5-11.	Basilicata
285	Pieroni, A.; Nebel, S.; Quave, C.; Munz, H.; Heinrich, M. Ethnopharmacy of the ethnic Albanians of northern Basilicata, Italy. *Fitoterapia* **2002,** *73* , 217-241.	Basilicata
286	Pieroni, A.; Nebel, S.; Quave, C.; Münzc, H.; Heinrich, M. Ethnopharmacology of liakra, traditional weedy vegetables of the Arbëreshë of the Vulture area in southern Italy. *J. Ethnopharmacol.* **2002,** *81* , 165-185.	Basilicata
287	Pieroni, A.; Nebel, S.; Santoro, R.C.; Heinrich, M. Food for two seasons: culinary uses of non-cultivated local vegetables and mushrooms in a south Italian village. *Int. J. Food Sci. Nutr* . **2005,** *56* , 245–272.	Basilicata
288	Pieroni, A.; Quave, C.L. Functional food or food medicine? On the consumption of wild plants among Albanians and Southern italians in Lucania. In *Eating and healing: traditional food as medicine* ; Pieroni, A., Price, L.L., Eds.; Haworth Press: Binghamton, New York, 2006; pp. 101–129.	Basilicata

289		Pieroni, A.; Quave, C.L.; Santoro, R.C. Folk pharmaceutical knowledge in the territory of the Dolomiti Lucane, inland southern Italy. *J. Ethnopharmacol.* **2004,** *95* , 373–384.	Basilicata
290		Pignone, D.; Laghetti, G. On sweet acorn (Quercus spp.) cake tradition in Italian cultural and ethnic islands. *Genet Resour Crop Evol* . **2010,** *57* , 1261–1266.	Sardinia
291		Pinna, C. Contributo allo studio e valorizzazione del pane di ghiande Sardegna. *Medit.* **2015,** *37* , 44-52.	Sardinia
292		Piras, G. Elementi della dendroflora sarda impiegati nella tradizione popolare di Putifigari e Villanova Monteleone (Sardegna Nord-Occidentale). *Cultural heritage and sustainable forest management: the role of traditional knowledge* **2006,** 506-516.	Sardinia
293		Poggio, L. *Piante officinali della Valle d'Aosta* ; Ed. Neos-Tipolito Subalpina 2006; pp. 108.	Aosta Valley
294		Quave, C.L.; Pieroni, A. Traditional health care and food and medicinal plant use among historic Albanian migrants and Italians in Lucania, Southern Italy. In *Traveling cultures and plants. The ethnobiology and ethnopharmacy of human migrations;* Pieroni, a., Vandebroek, I, Eds.; Berghahn: New York, 2007; pp. 204–227.	Basilicata
295		Raimondo, F.M. Le piante della flora locale nella tradizione popolare delle Madonie. *Giorn. Bot. Ital.* **1980,** *114,* 142.	Sicily
296		Raimondo, F.M.; Lentini, F. Indagini etnobotaniche in Sicilia. I. Le piante della flora locale nella tradizione popolare delle Madonie (Palermo). *Naturalista siciliano (Palermo)* **1990,** s. IV, *3-4* , 77-99.	Sicily
297		Ranfa, A.; Bodesmo, M. An Ethnobotanical investigation of traditional knowledge and uses of edible wild plants in the Umbria Region, Central Italy. *J. Appl. Bot. Food Qual.* **2017,** *90* , 246-258.	Umbria
298		Ranfa, A.; Maurizi, A.; Romano, B.; Bodesmo, M. The importance of traditional uses and nutraceutical aspects of some edible wild plants in human nutrition: the case of Umbria (central Italy). *Plant Biosyst.* **2014**, *148,* 297-306.	Umbria
299		Renna, M. Wild edible plants as a source of mineral elements in the daily diet. *Progr. Nutr.* **2017,** *19* , 219-222.	Italy
300		Renna, M.; Cocozza, C.; Gonnella, M.; Abdelrahman, H.; Santamaria, P. Elemental characterization of wild edible plants from country side and urban areas. *Food Chem* . **2015,** *177* , 29-36.	Apulia
301		Riccardo S. *Le piante spontanee eduli;* Ed. Battiato: Catania, Italy, 1921.	Italy
302	*	Rieppi, A. *Piante mangerecce spontanee del Friuli;* Comitato Nazionale Forestale, Italy, 1941.	Friuli Venezia Giulia
303		Rinaldi, A. Nomi volgari in uso nella valle di Ussita (Macerata). *Giorn. Bot. Ital.* **1965,** *72* (4-6), 680-682.	Marche
304		Rodato, S. *Piante spontanee utilizzate in alimentazione;* G.S. Stampa: Caselle d'Asolo (TV), Italy, 1989.	Veneto
305		Rolli, E.; Belli, B.; Bianchi, A. Le food plants in Toscana: tradizione e scienza nutraceutica. *Natural* **2014,** *1* , 1-47.	Tuscany
306		Rosati, A. Le più note piante spontanee di stagione: il chenopodio (Chenopodium album). *Vita in campagna* **2005**, *6,* 59-61.	Italy
307	*	Rovesti, G. *Le piante aromatiche e medicinali spontanee della Provincia di Porto Maurizio;* I Ed. 1922, Ed. Zem, 2010.	Liguria
308		Salerno, G.; Guarrera, P.M. Ricerche etnobotaniche nel Parco Nazionale del Cilento e Vallo di Diano: il territorio di Castel San Lorenzo (Campania, Salerno). Inf. Bot. Ital. 2008, 40 (2), 165-181.	Campania
309		Salerno, G.; Guarrera, P.M.; Caneva, G. Agricultural domestic and handicraft folk uses of plants in the Thyrrenian sector of Basilicata (Italy). *J. Ethnobiol. Ethnomed.* **2005**, *1* :2.	Basilicata
310		Sansanelli, S.; Ferri, M.; Salinitro, M.; Tassoni, A. Ethnobotanical survey of wild food plants traditionally collected and consumed in the Middle Agri Valley (Basilicata region, Southern Italy). *J. Ethnobiol. Ethnomed.* **2017**, *13:* 50	Basilicata
311		Sansanelli, S.; Tassoni, A. Wild food plants traditionally consumed in the area of Bologna (Emilia Romagna region,Italy). *J. Ethnobiol. Ethnomed.* **2014,** *10:* 69	Emilia Romagna
312		Savo, V. *Usi delle piante in costiera amalfitana. Uses of plants in the Amalfi coast.* Ed. Officine Zephiro: Amalfi, Italy, 2010; pp. 107.	Campania
313		Savo, V.; Caneva, G.; Guarrera, P.M.; Reedy, D. Folk phytotherapy of the Amalfi Coast (Campania, Southern Italy). *J. Ethnopharmacol* . **2011,** *135* , 376–392.	Campania

314	Savo, V.; Guarrera, P.M. Comprensorio Tolfetano-Cerite-Manziate. Specie vegetali tipiche di uso popolare. Ed. Gangemi, Rome, 2010.	Latium
315	Savo, V.; Salomone, F.; Bartoli, F.; Caneva, G. When the local cuisine still incorporates wild food plants: the unknown traditions of the Monti Picentini Regional Park (Southern Italy). *Economic Botany* **2019,** *73* (1), 28-46.	Campania
316	Scherrer Avalla, M.; Motti, R.; Weckerle Caroline, S. Traditional plant use in the areas of Monte Vesole and Ascea, Cilento National Park (Campania, Southern Italy). *J. Ethnopharmacol* . **2005**, *97,* 129–143.	Campania
317	Schicchi, R.; Geraci, A. Verdure Spontanee di Sicilia. Guida al Riconoscimento, alla Raccolta e alla Preparazione; Ed. IDIMED: Palermo, Italy, 2016.	Sicily
318	Scortegagna, S. *Flora Popolare Veneta - Nomi e usi tradizionali delle piante nel Veneto. Ed.* WBA Monographs 3: Verona, Italy, 2014; pp. 704.	Veneto
319	Sella, A. *Flora popolare biellese. Nomi dialettali, tradizioni e usi locali* . Collana della Fondazione Sella. Ed. Dell'Orso: Alessandria, Italy, 1992.	Piemonte
320	Selva, A.M. Piante officinali nella medicina popolare del Carso Isontino. *Herbora,* Atti delle giornate 23-38 Maggio 1979, Verona; pp. 334-337.	Friuli Venezia Giulia
321	Servettaz, O.; Colombo, M. L.; Banfi, E. Osservazioni sull'uso delle piante officinali nel Medio Novarese. *Atti dell'Istituto Botanico dell'Università e Laboratorio Crittogamico Pavia* **1979,** *XIII* , 181-202.	Piemonte
322	Signorello, P. *Piante Spontanee Siciliane Mangerecce e Velenose;* Stampa Massimino, 2015; pp. 191.	Sicily
323	Signorini, M.A.; Lombardini, C.; Bruschi, P.; Vivona, L. Conoscenze etnobotaniche e saperi tradizionali nel territorio di San Miniato (Pisa). *Atti Soc. tosc. Sci. nat. Mem* . **2007,** Serie B, *114,* 65-83.	Tuscany
324	Signorini, M.A.; Piredda, M.; Bruschi, P. Plants and traditional knowledge: An ethnobotanical investigation on Monte Ortobene (Nuoro, Sardinia). *J. Ethnobiol. Ethnomed.* **2009,** *5:* 6.	Sardinia
325	Taffetani, F. *Rugni Speragne e Crispigne. Piante spontanee negli usi e nelle tradizioni del territorio maceratese* . Fondazione Cassa di Risparmio della Provincia di Macerata, 2005.	Marche
326	Tammaro, F. *Flora Officinale d'Abruzzo Giunta Regionale d'Abruzzo* . Centro Servizi Culturali, Chieti, 1984.	Abruzzi
327	Tammaro, F.; Pietrocola, L. Usi popolari delle piante in Abruzzo. *Quaderni della Rivista Abruzzese* **1975,** *4,* 1-56.	Abruzzi
328	Tomei, P. E.; Maccioni, S.; Parmigiani, M. L'uso medicinale delle piante nella tradizione popolare della Lunigiana. Il contributo. *Mem. Acc. Lunigianese Sci. G. Cappellini, Sci. Mat., fis. e nat.* **1997/1999**, *67-69,* 209-221.	Tuscany
329	Tomei, P.; Camangi, F. La "cucina" carrarese e messese: aspetti botanici e alimurgici. *Paralleli e Meridiani* **2004,** *2 (* 1), 55-66.	Tuscany
330	Tomei, P.; Camangi, F. *Tradizioni alimurgiche in Toscana . Piante selvatiche e coltivate nella preparazione delle zuppe;* Ed. Maria Pacini Fazzi, Lucca, Italy, 2014.	Tuscany
331	Tomei, P.E.; Gaspari G. Indagine sulle zone umide della Toscana. XVI. LE piante officinali dei bacini palustri della Toscana Settentrionale. *Atti Soc. Tosc. Nat., Mem* ., Serie B **1981,** *88,* 175-194.	Tuscany
332	Tomei, P.E.; Lippi, A.; Uncini Manganelli, R.E. L'uso delle specie vegetali spontanee nella preparazione delle zuppe di magro in Lucchesia (LU). In *Funghi, tartufi ed erbe mangerecce.* Convegno Internazionale. Atti. L'Aquila 28 settembre, 1 ottobre. Accademia Italiana della cucina e Università degli studi de l'Aquila 1996; pp. 243.	Tuscany
333	Tomei, P.E.; Monti, G.; Onnis, A. *Specie vegetali coltivate e spontanee di uso alimentare e medicinale nella tradizione popolare dell'alta Garfagnana* . Dipartimento di Scienze Botaniche dell'Università di Pisa. Ed. Pacini: Pisa, Italy, 1988; pp. 48.	Tuscany
334	Tomei, P.E.; Trimarchi, S. *Piante d'uso etnobotanico in Toscana;* Ed Maria Pacini Fazzi, 2017; pp. 396.	Tuscany
335	Tomei, P.E.; Uncini Manganelli, R.E. (a cura di) Etnobotanica nel padule di Fucecchio 2005 http://www.parchiagricoltura.it/news.html	Tuscany
336	Uncini Manganelli, R.E., Camangi, F., Tomei, P.E. *L'uso delle erbe nella tradizione rurale della Toscana;* ARSIA Regione Toscana: Firenze, Italy, 2007; Volumes 1–3.	Tuscany

337	Uncini Manganelli, R.E.; Camangi, F.; Tomei, P.E. Primi appunti su alcune tradizioni alimentari nel territorio di Massaciuccoli (LU). In *Il Bacino del Massaciuccoli* . Ed. Pacini: Pisa, Italy, 1999; pp. 49-53.	Tuscany
338	Uncini Manganelli, R.E.; Tomei, P.E Indagini farmaco-botaniche in Garfagnana (Lucca): il versante apuano. *Atti Soc. Tosc. Sci. Nat., Mem ., Serie B* **1996**, *103* , 63-80.	Tuscany
339	Uncini Manganelli, R.E.; Tomei, P.E. *Indagini etno-farmacobotaniche nei Colli Pisani: da Guasticce a Palaia;* Dipartimento di Scienze Botaniche dell'Universita: Pisa, Italy, 1997; pp. 86-106.	Tuscany
340	Uncini Manganelli, R.E.; Tomei, P.E. Indagini etno-farmacobotaniche nel Pistoiese. *Atti Mem. Accad. Tosc. Sci. Lett. Colombaria* **1998**, *63* , N.S. 49, 135-158.	Tuscany
341	Uncini Manganelli, R.E.; Tomei, P.E. Indagini farmaco-botaniche in Garfagnana (Lucca): il versante appenninico. *Atti Soc. Tosc. Sci. Nat., Mem., Serie B* **1995**, *102* , 3-18.	Tuscany
342	Uncini Manganelli, R.E.; Tomei, P.E. *Le piante selvatiche nell'alimentazione e nella medicina popolare dei Colli Pisani;* ETS: Pisa, Italy, 1999.	Tuscany
343 *	Vaglio, M. *Erbe delle valli Alpine. Alla scoperta di 260 piante commestibili* ; Priuli & Verlucca: Scarmagno (TO), Italy, 2014; pp. 253.	Italy
344	Vallariello, G. *Etnobotanica dell'isola d'Ischia* (Napoli, Italia). *Delpinoa* **2003**, *45* , 233–243.	Campania
345	Vannelli, S. *Erbe selvatiche e commestibili della Sardegna* ; S.l., AM&D: Cagliari, Italy, 1998; pp. 192	Sardinia
346	Vanzani, P.; Rossetto, M.; De Marco, V.; Sacchetti, L.E.; Paoletti, M.G.; Rigo, A. Wild Mediterranean plants as traditional food: A valuable source of antioxidants. J. Food Sci. 2011, 76(1), C46–C51.	Mediterranean
347	Veri, L. Flora utile dei Monti Simbruini. *Micologia e Vegetazione mediterranea* **1997**, *XII* suppl. 1.	Latium
348	Vitalini, S.; Iriti, M.; Puricelli, C.; Ciuchi, D.; Segale, A.; Fico, G. Traditional knowledge on medicinal and food plants used in Val San Giacomo (Sondrio, Italy)—An alpine ethnobotanical study. Journal of Ethnopharmacology, 2013, 145 (2), 517-529.	Lombardia
349	Vitalini, S.; Puricelli, C.; Mikerezi, I.; Iriti M. Plants, people and traditions: Ethnobotanical survey in the Lombard Stelvio national park and neighbouring areas (central Alps, Italy). Jo*urnal of Ethnopharmacology* **2015**, *173* , 435-458.	Lombardia
350	Vitalini, S.; Tomè, F.; Fico, G. Traditional uses of medicinal plants in Valvestino (Italy) *Journal of Ethnopharmacology* **2009**, *121* : 106–116.	Lombardia
351	Zampiva, F. *Erbario veneto: cultura, usi e tradizioni delle piante e delle erbe più note;* Egida Ed.: Vicenza, Italy, 1999.	Veneto
352	Zampiva, F. Erbe e piante della Lessinia. *Erboristeria domani* **1981**, *9* , 17-25.	Veneto
353	Zampiva, F. Etnobotanica ed etnomedicina: le erbe, le piante e i loro usi in Lessinia. In AA. VV. *Civiltà Cimbra;* Bi & Gi ed.: Verona, Italy, 1983; pp. 175-183.	Veneto
354	Zampiva, F. Le erbe nella cucina popolare veneta. *Erboristeria Domani* **1983,** *4* , 60-69.	Veneto
355	Zanin, G.; Zuin, M.C.; Vigolo, M. T.; Zanin, G. *Il giardino fitoalimurgico per la valorizzazione delle piante spontanee;* Veneto Agricoltura: Legnaro (PD), Italy, 2008.	Veneto
356	Zanotti, E.; Gorno, G.; Paletti, G. Le piante selvatiche commestibili con note sulle loro proprietà medicinali e ricette di cucina. *I quaderni del Parco* **2012**, *9*.	Lombardia
357	Zuin, M.C. *Il giardino fitoalimurgico per la valorizzazione delle piante spontanee;* Veneto Agricoltura: Legnaro (PD), Italy, 2010.	Veneto
358	Zuin, M.C.; Zanin, G.; Zanin, G. Il giardino fitoalimurgico per la valorizzazione delle piante spontanee. *Agribusiness Paesaggio & Ambiente* **2006**, *IX (* 1), 75-82.	Veneto
